# A comprehensive map of missense trafficking variants in rhodopsin and their response to pharmacologic correction

**DOI:** 10.1126/sciadv.aef3518

**Published:** 2026-07-29

**Authors:** Kannan V. Manian, Connor H. Ludwig, Yan Zhao, Nathan S. Abell, Robert Warneford-Thomson, Henry Chan, Zachary O. Casey, Xiaoping Yang, David E. Root, Matthew L. Albert, Jason Comander

**Affiliations:** ^1^ Ocular Genomics Institute, Berman-Gund Laboratory for the Study of Retinal Degenerations, Mass Eye and Ear, Harvard Medical School, Boston, MA, USA.; ^2^ Octant, Inc., Emeryville, CA, USA.; ^3^ Molecular Biology Institute, University of California, Los Angeles, CA, USA.; ^4^ Genetic Perturbation Platform, Broad Institute of MIT and Harvard, Cambridge, MA, USA.

## Abstract

Rhodopsin (*RHO*) missense variants are a leading cause of autosomal dominant retinitis pigmentosa (adRP), a progressive retinal degeneration. Interpreting *RHO* variant pathogenicity is challenging, and understanding their disease mechanisms is essential for developing therapeutics. We present a high-resolution map of *RHO* missense variant trafficking using deep mutational scanning approaches, including a surface abundance immunoassay and a complementary membrane proximity assay. This comprehensive, reproducible dataset encompassed all 6612 possible missense variants. Over 700 variants had pathogenic trafficking scores, substantially expanding the number of *RHO* variants with functional data. Trafficking scores correlated with the magnitude of ER stress markers and ClinVar pathogenicity classifications. Data also identified structurally clustered mutational intolerance around the intradiscal beta-plug region. Treatment with the chaperone YC-001 restored surface trafficking in most mistrafficking variants. This functional map of *RHO* variants provides a valuable resource for pathogenicity assessment, genotype-phenotype correlations, and the development of targeted therapeutic strategies for *RHO*-adRP.

## Teaser

An atlas of rhodopsin missense variants maps their misfolding potential, pathogenicity, and therapeutic drug rescue.

## INTRODUCTION

The rhodopsin (*RHO*) gene holds a unique place in genetics and structural biology. It was among the first human disease genes identified using modern molecular methods, and the first gene identified to cause an inherited retinal degeneration (*1*–*3*). As a G-protein-coupled receptor (GPCR) essential for rod photoreceptor function, it was also the first GPCR to be crystallized, revealing the now-familiar 7-transmembrane structure (*4*). This provided a foundational model for GPCR structure-function relationships, influencing drug discovery across diverse therapeutic areas (*5*, *6*). More than three decades of research, spanning mutagenesis studies (*7*), biophysical characterization (*8*), and extensive structure-function analyses (*9*), have shaped our understanding of RHO’s function and its role in the progressive, Mendelian retinal disease autosomal dominant retinitis pigmentosa (*RHO*-adRP) (*10*, *11*). Despite these advances, a comprehensive map of RHO missense variants and their functional consequences remains elusive. Deep mutational scanning (DMS) has emerged as a transformative approach for systematically mapping structure-function relationships and measuring disease-associated phenotypes across entire mutational landscapes (*12*). This approach has been successfully applied to proteins with well-defined folding and trafficking pathways, including ion channels (*13*, *14*), tumor suppressors (*15*, *16*), and metabolic enzymes (*17*, *18*), to resolve variant pathogenicity (*19*–*21*) and identify therapeutic entry points (*20*). Given rhodopsin’s central place in vision research, the genetic heterogeneity of *RHO*-adRP, and the emergence of clinical trials for RHO-adRP patients, a systematic characterization of *RHO* variants is increasingly needed.

Pathogenic RHO variants that cause *RHO*-adRP span multiple mechanistic classes, with misfolding and endoplasmic reticulum (ER) retention representing the most prevalent pathogenic mechanism. This class of variants, designated “Class 2”, disrupts rhodopsin’s trafficking through the secretory pathway, leading to ER stress and photoreceptor cell death (*10*, *22*). While some functional data exist for well-characterized variants, most RHO variants remain unclassified mechanistically; in fact, for most of the RHO missense variants detected by genetic testing, it is unknown whether they even cause disease (labeled as a Variant of Uncertain Significance [VUS] in the ClinVar database, https://www.ncbi.nlm.nih.gov/clinvar/, accessed September 2025). This lack of information limits genetic counseling, prognostic assessment, and targeted therapeutic development (*23*). As a potential strategy to address this need, the American College of Medical Genetics and Genomics (ACMG) scoring system for variant pathogenicity allows for well-validated functional assays to contribute to pathogenicity classification (*24*–*27*). Prior studies have successfully used heterologous cells to demonstrate cell trafficking defects of RHO variants, including a set of 210 literature and clinical RHO variants (*28*), a set of 123 pathogenic RHO variants (*29*), and 700 transmembrane domain RHO variants (*30*). However, no systematic effort has comprehensively resolved the functional consequences of all RHO missense variants. A high-resolution functional landscape of RHO variants would enable pathogenicity assessment, inform genotype-phenotype correlations, and provide a foundation for previously unexplored therapeutic strategies.

Herein, we apply two complementary DMS approaches to systematically profile the trafficking properties of all possible RHO missense variants, generating a comprehensive functional map of RHO mistrafficking variants due to misfolding. We identify structurally clustered, trafficking-deficient variants enriched in pathogenic annotations and demonstrate that a previously reported small-molecule corrector, YC-001 (*31*), can rescue the majority of these variants. These data enhance our understanding of rhodopsin biology, provide a scalable framework for variant classification, and lay the foundation for precision ophthalmology approaches to *RHO*-adRP. By integrating decades of research findings with modern high-throughput functional genomics, this approach represents a strategy for accelerating genetic discovery and therapeutic interventions, expanding opportunities for individualized treatment strategies in inherited retinal degeneration diseases.

## RESULTS

### Establishment of dual deep mutational scanning assays for RHO surface trafficking

**Fig. 1.**
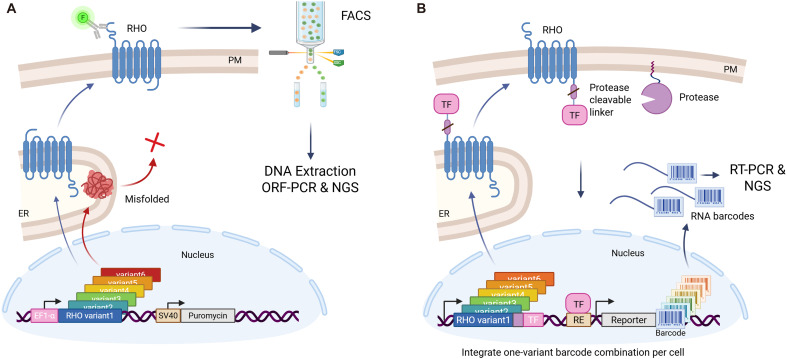
Dual deep mutational scanning assays enable high-resolution measurements of RHO variant surface trafficking. (**A**) In Method 1, a single-site saturation mutagenesis library of *RHO* is transduced into HEK293T cells. *RHO* variants are constitutively expressed in their native, untagged form, and cells are selected using puromycin. Cells are fixed, immunostained with a fluorophore-conjugated antibody that recognizes the extracellular N- terminus of RHO, and subjected to fluorescence-activated cell sorting (FACS) to separate and sort the top 20% RHO^High^ expression bin and the bottom 20% RHO^Low^ expression bin. Genomic DNA from each population is reverse-crosslinked, RHO variant sequences are amplified with open reading frame polymerase chain reaction (ORF-PCR), and variant frequencies in each population are quantified using next generation sequencing (NGS). (**B**) In Method 2, a single-site saturation mutagenesis library of *RHO* is integrated at single copy into a specific landing pad in the *H11* safe harbor locus of HEK293T cells. RHO variants are inducibly expressed using the Tet-ON system and are expressed with a C-terminally fused protease-cleavable linker followed by a transcription factor (TF). Proper folding of RHO in the endoplasmic reticulum (ER) and trafficking to the plasma membrane (PM) positions it proximal to a membrane-anchored protease that releases the TF to translocate into the nucleus, thus allowing binding to a specific DNA response element (RE) and initiation of transcription of a variant-specific barcode. RNA barcodes are purified from cell lysates and used in a reverse transcription reaction to make complementary DNA barcodes, which are amplified by PCR and read out by NGS. Created in BioRender. Manian, K. (2026) https://BioRender.com/rifnazn.

To systematically identify single-residue RHO variants with folding and trafficking defects, we developed two complementary, highly scalable, multiplex, HEK293Tcell–based assays capable of measuring RHO surface trafficking. The first method, henceforth Method 1, used a flow cytometry-based immunoassay to quantify trafficking and steady-state surface abundance of RHO (Fig. 1A). Briefly, cells were transduced with a lentiviral library (fig. S1A) containing all single-residue missense, nonsense, and synonymous RHO variants, using a low multiplicity of infection to ensure integration of only one variant per cell. Approximately 500 cells per variant were transduced to buffer against cell-to-cell variability in the lentiviral integration site. Successfully transduced cells, constitutively expressing RHO, were selected with puromycin. For the assay, cells were fixed and stained with an antibody recognizing approximately the first 10 residues of the extracellular N-terminus of RHO. Cells were then subjected to fluorescence-activated cell sorting (FACS) for separation into high- and low-fluorescence bins (see Methods). Genomic DNA from each bin was isolated, amplified, and RHO variants were read out by next-generation sequencing (NGS) and quantified.

The second method, henceforth “Method 2”, used a transcriptional reporter assay to measure surface trafficking of RHO (Fig. 1B). Cells harboring a landing pad in the *H11* locus were co-transfected with a plasmid library containing all single-residue RHO missense and nonsense variants as well as a plasmid encoding the Bxb1 integrase. This integrase ensured landing pad-specific, single-copy integration of an all-in-one library construct (fig. S1B) containing the following elements: (i) a constitutively expressed plasma membrane-anchored protease, (ii) one inducibly (Tet-On) expressed *RHO* variant fused via a protease-cleavable linker to a transcription factor, and (iii) a reporter gene that produced a unique RNA barcode upon transcription factor binding that served as an identifier for the encoded genetic variant. Proper trafficking of RHO to the plasma membrane resulted in protease-mediated release of the transcription factor, which, once free, translocated to the nucleus and activated transcription of the DNA-encoded RNA barcode. RNA was harvested from cells and converted to cDNA, and the transcribed barcodes were sequenced and quantified by NGS.

For both methods, sequencing counts were modeled using a negative binomial generalized linear mixed-effects model (NBGLMM) to estimate variant effects. Effect sizes describing the log_2_ fold change between variant and wild-type were adjusted to a zero-to-one log scale, where zero represents the global mean of nonsense variant surface trafficking scores and one represents wild-type or synonymous variant scores.

### Variant trafficking scores correlate between complementary DMS assays

**Fig. 2.**
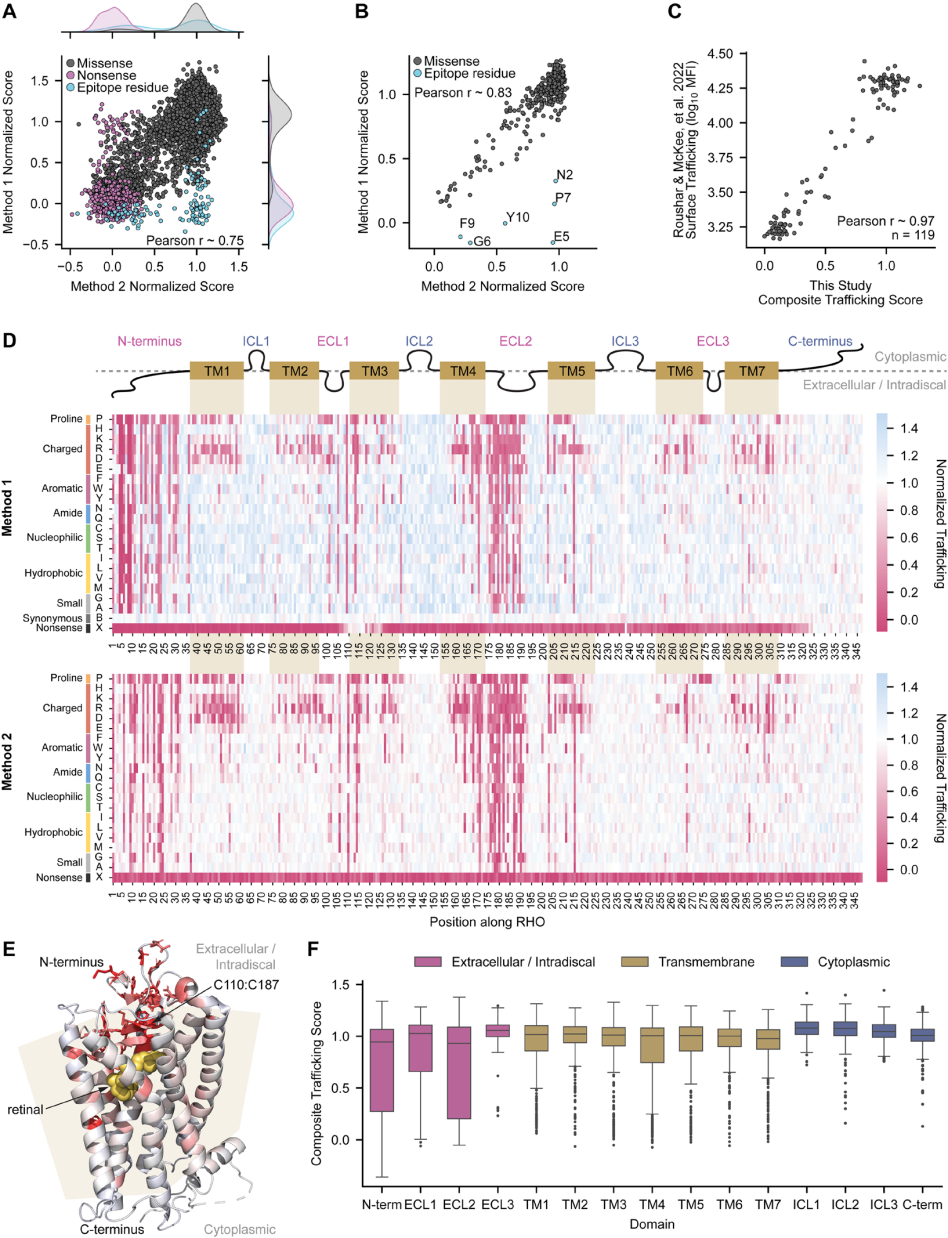
Comprehensive assessment of RHO missense variants reveals that extracellular residues above the retinal-binding pocket are particularly mutationally intolerant. (**A**) Scatterplot showing strong agreement between deep mutational scanning (DMS) scores for Method 1 (*y*-axis) and Method 2 (*x*-axis) at the variant level, with adjunct kernel density estimations showing the distribution of scores for each dataset. Variants affected by Method 1- and Method 2-specific artifacts are highlighted in blue and pink, respectively (Pearson’s *r* = 0.75). (**B**) Scatterplot showing strong agreement between average DMS scores at the position (residue) level. Nonsense variants (affected by Method 2-specific artifacts) are excluded, and variants affected by Method 1-specific artifacts are highlighted in blue (Pearson’s *r* = 0.83). (**C**) Scatterplot showing strong agreement between the composite trafficking scores from this study and surface trafficking measurements from a flow cytometry-based HEK293T immunoassay (N = 119 variants, *r* = 0.97) (*29*). (**D**) Heatmaps showing surface trafficking for Method 1 (top) and Method 2 (bottom) for all missense and nonsense variants. The *x*-axis indicates the position along the RHO protein, with domain boundaries indicated (TM = transmembrane, ICL = intracellular (cytoplasmic) loop, ECL = extracellular (intradiscal) loop). The *y*-axis shows the amino acid substituted at each position. RHO surface trafficking is indicated by cell color. White: trafficking similar to wild-type; pink: a trafficking defect relative to wild-type; blue: a trafficking increase relative to wild-type. Missing measurements are indicated in gray. Values are normalized on a log scale from 0 (mean of non-trafficking, nonsense variant negative controls) to 1 (wild-type RHO surface trafficking, positive control). (**E**) RHO structure (PDB: 1F88) colored according to the average composite DMS score (from meta-analysis, see Methods) at each position. The natural ligand, retinal, is indicated in yellow (for illustrative purposes only). (**F**) Distributions of composite trafficking scores for each domain of RHO.

For Method 1, we performed a single library transduction and two replicate FACS assays. We recovered 6572 of the 6612 missense variants (99.4%), 346 of the 348 nonsense variants, and 328 of the 348 synonymous variants. For Method 2, we performed a single library transfection and eight replicate assays. We recovered 6611 of the 6612 missense variants (>99.9%) and all 348 of the nonsense variants, with a median of 57.5 unique barcodes per variant per RNA-seq sample. For both methods, surface trafficking data were bimodally distributed (Fig. 2A), with 1046 (15.9%) and 1238 (18.7%) missense variants exhibiting significantly lower surface trafficking than wild-type in Method 1 and Method 2, respectively, at a 5% FDR (data file S1).

The use of two complementary DMS approaches provided an opportunity to assess RHO trafficking with independent methodologies. Despite the methodological distinctions between Method 1 and Method 2 (Fig. 1), we observed strong agreement between Method 1 and Method 2 trafficking scores on a per-variant level (Fig. 2A Pearson *r* = 0.75, *P* < 0.01) and on a per-residue level (Fig. 2B, Pearson *r* = 0.83, *P* < 0.01). Moreover, the use of complementary methodologies enabled us to fill gaps where technical limitations affected one assay but not the other. Notably, the most discordant variants were within the N-terminal, surface-exposed epitope region (residues 2–10), which interfered with antibody binding and accounted for the observed low signal for Method 1 (Fig. 2, A and B, blue). By contrast, the use of a transcription factor fused to the C-terminus of RHO in Method 2 accounted for the inability to detect truncation variants that were nonetheless capable of trafficking to the cell surface (Fig. 2A, pink). Method 1 produced synonymous variant trafficking scores at every position in the library, and they demonstrated a strong separation from the scores of nonsense variants. While the vast majority of nonsense variants failed to traffic to the plasma membrane, Method 1 revealed a subset of nonsense variants from residues 110–120 (after the first two transmembrane domains) and 320–348 (unstructured cytosolic C terminus) that had near-normal surface trafficking. Consistent with the pooled assay results, immunofluorescence and flow cytometry analysis of individual variants confirmed the moderate surface trafficking for the nonsense variants G114* and F116*, but showed no detectable surface trafficking for a nonsense variant outside that region, W126* (fig. S2). Because these truncating variants are expressed from cDNA, they may have different expression levels when expressed from the endogenous locus, where many premature termination codons are usually expected to trigger nonsense-mediated decay and reduce or eliminate protein expression. Accordingly, apparent ‘normal’ surface trafficking for some truncating variants in this assay should be interpreted cautiously.

We next performed a meta-analysis using the Method 1 and Method 2 trafficking scores in order to obtain an integrated, precise trafficking metric for each variant. The variants with the top 5% most discordant scores were flagged (Methods, I^2^ heterogeneity threshold of 93). The composite trafficking scores strongly correlated with those measured by a flow cytometry-based HEK293T immunoassay (similar to Method 1) in a recent, independent study of 119 pathogenic RHO missense variants (*29*) (Fig. 2C, Pearson *r* = 0.97, *P* < 0.01), (data file S2).

### Mutational intolerance clusters spatially around the intradiscal beta-plug

Variants with surface trafficking defects were not evenly distributed across the RHO protein. Strikingly, a large fraction of substitutions within the N-terminal region and second extracellular loop (ECL2) produced severe trafficking defects (Fig. 2, D to F). These regions cover the retinal-binding pocket, harbor crucial residues for proper protein folding and glycosylation, and coordinate key interactions with ECL1 and ECL3 (*32*). For instance, all substitutions were deleterious at C110 and C187, which form a critical disulfide bridge between ECL1 and ECL2 (*33*). Additionally, the seven transmembrane structure of RHO is clearly visible in the heatmap, with intolerance to transmembrane (TM) domain substitutions with charged amino acids, which are electrostatically disfavored in the hydrophobic lipid bilayer (Fig. 2, D and E), and with proline, a canonical helix-breaker (*34*–*37*). Both DMS methods showed normal trafficking of missense and nonsense variants within the RHO C-terminal tail (residues 326–348) (Fig. 2, D to F), which contains an outer segment/ciliary targeting signal that, when altered, can disrupt RHO localization in photoreceptors (*38*–*40*). Because these measurements are made outside of a photoreceptor/polarized ciliary context, our HEK293T-based assays do not capture outer-segment targeting defects. Accordingly, additional assays will be needed to detect C-terminal mutations in a high-throughput manner.

### Trafficking scores show high concordance with established mechanistic classes

**Fig. 3.**
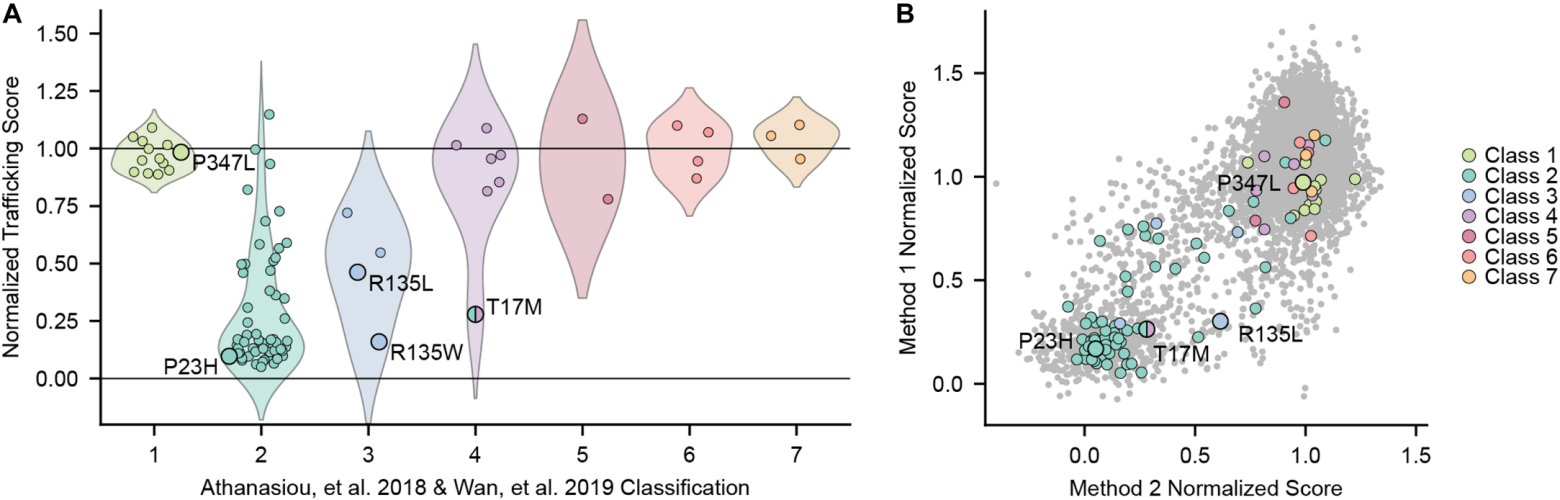
Variants with trafficking defects are enriched among the misfolding mechanistic class. (**A**) Distributions of the composite trafficking scores (*y*-axis) of literature-annotated variants stratified by their biochemical mechanistic class descriptions in Athanasiou *et al.* (*10*) and updated in Wan *et al.* (*28*). Composite trafficking scores are normalized from 0 (mean of non-trafficking negative controls) to 1 (wild-type surface trafficking, positive control). The biochemical mechanistic classes are as follows: (1) failure to localize to the outer segment, (2) misfolded and trapped in the ER, (3) defective endocytosis, (4) defective post-translational modification, (5) defective G protein activation, (6) constitutive activation, and (7) defective dimerization. Select variants are indicated. (**B**) Scatterplot showing trafficking scores from Method 1 (*y*-axis) and Method 2 (*x*-axis) at the variant level overlaid with variant classifications as reviewed in Athanasiou *et al.* (*10*) and updated in Wan *et al.* (*28*). Select variants are labeled: Class 1 - P347L, Class 2 - P23H, Class 3 - R135L, dual Class 2/4 - T17M.

Mechanistic class labels for RHO variants have developed over time, and one system defines seven mechanistic classes for pathogenic RHO variants based on their biochemical and cellular dysfunction: altered post-Golgi trafficking and outer segment targeting (Class 1), misfolding with ER retention and instability (Class 2), disrupted vesicular trafficking and endocytosis (Class 3), altered post-translational modifications and stability (Class 4), altered transducin activation (Class 5), constitutive activation (Class 6), and dimerization deficiency (Class 7) (*10*, *28*). Most pathogenic variants that have been assigned as Class 2 were classically assayed by measuring surface trafficking in cell culture (*28*, *41*–*43*). To assess the ability of both methods to identify surface trafficking defects for Class 2 variants, we overlaid the biochemical mechanistic class labels for the small set of 91 literature-annotated variants (*10*, *28*). As expected, annotated variants with the greatest surface trafficking defects belonged to Class 2, including the founder variant P23H (Fig. 3, A and B). While most annotated Class 2 variants mistrafficked, a few had no trafficking defects, suggesting consideration for reclassification. We observed a range of trafficking scores for Class 3 variants, which have only been described at R135 (Fig. 3A). R135W, the Class 3 variant with the most severe trafficking defect, has been described as misfolded and ER-retained, which are hallmarks of Class 2 (*44*)(Fig. 3A). Misfolding and ER retention have not previously been observed for R135L and R135P, which had some of the most discordant scores between Method 1 and Method 2 that may reflect differences in the kinetics of RHO trafficking between the methods. Aside from T17M, which is an annotated dual Class 2 and Class 4 variant, no Class 1, 4, 5, 6, or 7 variants exhibited clear surface expression defects (Fig. 3, A and B), pointing to the specificity of both methods in measuring trafficking defects for Class 2 and, to a lesser extent, Class 3 mechanistic classes.

### Variants with trafficking defects cause misfolding and ER stress

**Fig. 4.**
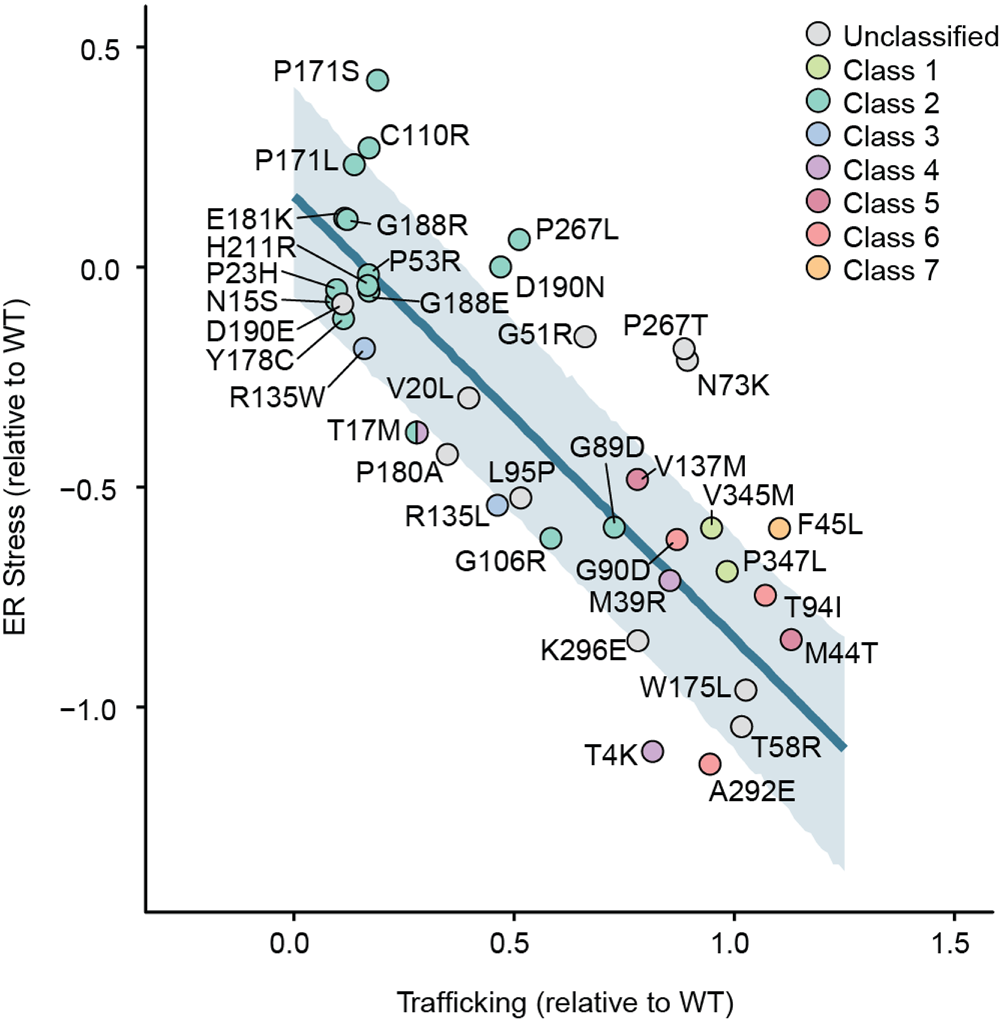
Variants with trafficking defects exhibit higher levels of ER stress. Levels of ER stress, measured by RT-qPCR expression of spliced unfolded protein response gene *XBP1* in a transgenic cell model, are displayed against the composite trafficking score for a selected panel of 38 variants. The ER stress data for each variant is expressed as the log_2_ fold change relative to cells expressing WT RHO, which is 0. The blue line and shaded region represent the fitted meta-regression model and 95% credible interval, respectively (see Methods). Variants are colored by the biochemical mechanistic class as previously classified in Athanasiou *et al.* (*10*) and updated in Wan *et al.* (*28*), as described above.

Class 2 variants are known to misfold and accumulate in the ER, causing ER stress and activating the unfolded protein response (UPR) pathways (*10*). UPR activation triggers the downstream sensor IRE1α to drive the splicing of *XBP1* (X-box binding protein 1) mRNA to mitigate ER stress (*45*–*47*). We hypothesized that variants with greater mistrafficking would exhibit higher ER stress levels. To test this, we selected 38 pathogenic variants spanning a range of trafficking scores and annotated mechanistic classes, generated doxycycline-inducible HEK293T stable cell lines expressing each variant, and quantified *XBP1* mRNA abundance upon RHO variant expression. We observed that variants with lower surface trafficking exhibited higher ER stress, a relationship confirmed by Bayesian meta-regression analysis that revealed a strong negative association (β = −1.00, 95% CI: −1.21 to −0.82) (Fig. 4). Overall, this relationship between trafficking score and *XBP1* expression supports the hypothesis that trafficking defects are caused by protein misfolding, and in turn UPR activation. Additionally, these data confirm that in this cell culture system, Class 1, 5, 6 and 7 variants have pathogenic mechanisms independent of ER stress. Furthermore, these data validate previous reports that the annotated Class 3 R135W variant is ER-retained and provides additional evidence supporting the dual Class 2 / Class 4 label for T17M.

### RHO trafficking robustly discriminates pathogenic variants with high specificity

**Fig. 5.**
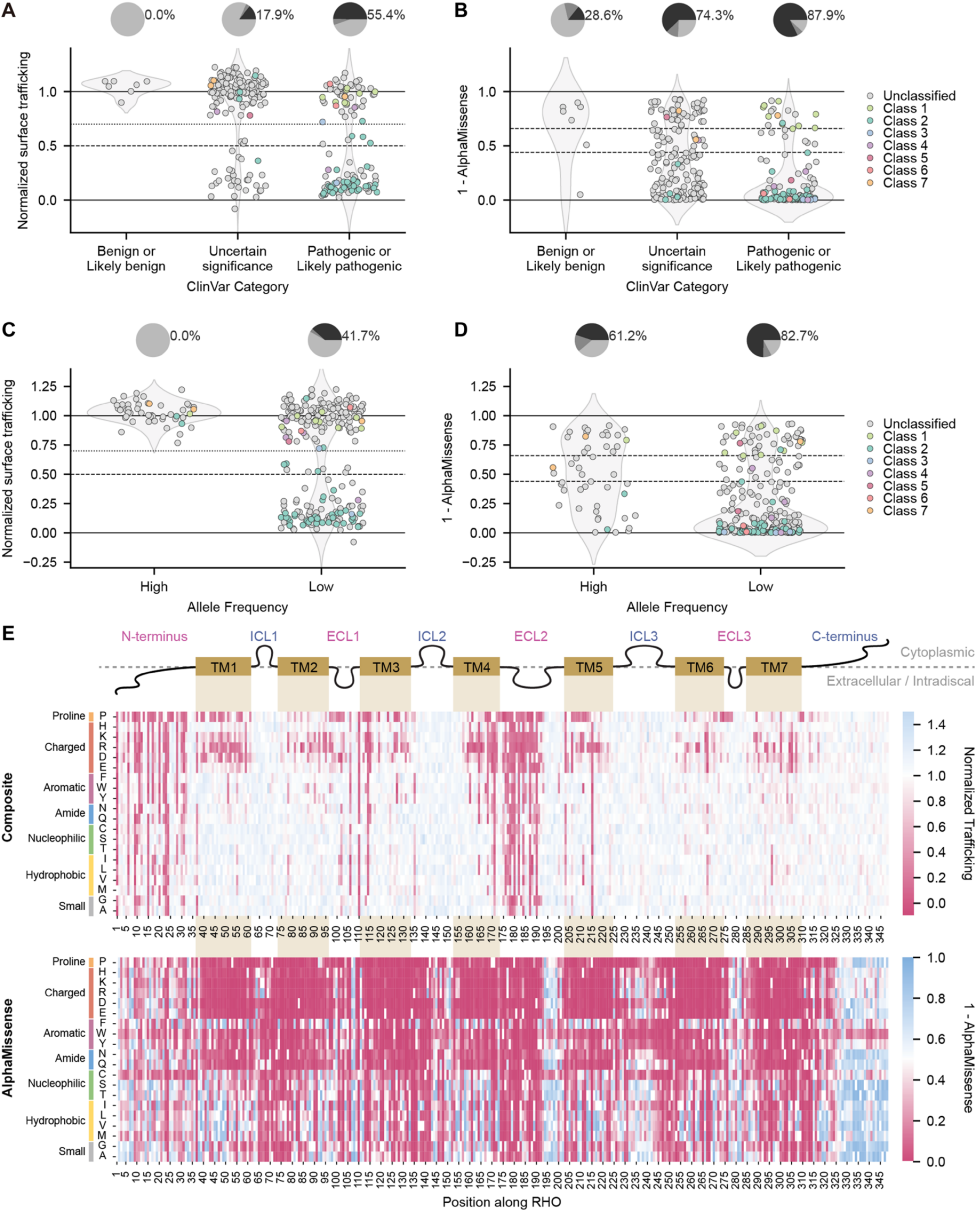
Trafficking defects predict variant pathogenicity. (**A**) Strip plot showing the composite normalized trafficking score for Method 1 and Method 2 for variants registered in the ClinVar database, stratified by clinical significance and colored by mechanistic classification as previously described in Athanasiou *et al.* (*10*) and Wan *et al.* (*28*). The dotted horizontal line at y = 0.7 represents the threshold for moderate confidence trafficking defects, while the dashed horizontal line at y = 0.5 represents the threshold for high confidence trafficking defects. Pie charts placed above each category show the percentage of variants with moderate or high confidence trafficking defects (light gray = no defect, medium gray = moderate confidence defect, dark gray = high confidence defect). (**B**) Strip plot showing one minus the AlphaMissense pathogenicity prediction score (0 = strong prediction of pathogenicity, 0.44–0.66 = uncertain prediction, 1 = strong prediction of benignity), stratified by clinical significance and colored by mechanistic classification, for the same set of variants as in (A). (**C**) Strip plot showing the composite normalized trafficking score, stratified by gnomAD allele frequency (threshold = 5 × 10^−5^) and colored by mechanistic classification, for the same set of variants as in (A). (**D**) Strip plot showing one minus the AlphaMissense pathogenicity prediction score, stratified by gnomAD allele frequency (threshold = 5 × 10^−5^) and colored by mechanistic classification, for the same set of variants as in (B). (**E**) Heatmaps showing composite trafficking scores (top) versus one minus prediction score from AlphaMissense pathogenicity prediction score (bottom).

Because the trafficking assay was primarily designed to identify Class 2 (misfolding and mistrafficking) RHO variants, we compared our assay results to ClinVar annotations to identify which known clinical variants had pathogenic trafficking levels. ClinVar is a well-established database of genetic variants categorized as benign, pathogenic, or VUS based on the body (or lack thereof) of functional, genetic, clinical, and other types of evidence (https://www.ncbi.nlm.nih.gov/clinvar/). First, we implemented numerical cutoffs for “moderate confidence” (<0.7) and “high confidence” (<0.5) surface trafficking defects (using the upper limit of the 95% confidence interval) as well as “low” (<0.5) and “very low” (<0.25) surface trafficking (using the mean) based on the composite trafficking score of known benign variants and Class 2 variants (fig. S3). Then, we compared our assay results with ClinVar annotations. Approximately 55% of variants in the combined pathogenic and likely pathogenic categories exhibited moderate or high confidence surface trafficking defects, whereas none in the combined benign and likely benign categories showed such defects (Fig. 5A). Thus, while a trafficking defect strongly suggests pathogenicity, a normal score does not exclude it, consistent with the existence of other mechanistic classes of mutations (*28*). In other words, the surface trafficking assays have excellent specificity and, as expected, moderate sensitivity when predicting overall pathogenicity. In this context, the subset of VUS that exhibit moderate or high confidence trafficking defects are now supported by orthogonal functional data, providing evidence that can contribute to their reclassification toward likely pathogenic or pathogenic categories (Fig. 5A) (*24*). Next, we calculated OddsPath values (*24*) for the composite trafficking score to formally estimate the statistical strength that the assay provides for variant pathogenicity classification. All 55 ClinVar-annotated benign/likely benign or common missense variants (gnomAD allele frequency > 5e-5) scored >0.7, while 91/140 (65%) of ClinVar-annotated pathogenic/likely pathogenic missense variants scored <0.7. The resulting OddsPath_abnormal value of 36 and OddsPath_normal value of 0.004 (see Methods) correspond to “strong” evidence supporting variant classification (strong PS3 and strong BS3, respectively). Given the assay’s mechanism and imperfect sensitivity, we interpret normal trafficking results cautiously and do not recommend applying a strong BS3 code based on this assay (see Discussion).

Overall, out of all possible 6612 RHO missense variants, 757 (11.4%) had moderate or high confidence trafficking defects (data file S1), substantially increasing the number of variants with evidence supporting pathogenicity compared to the 198 likely pathogenic or pathogenic variants of any type currently in ClinVar.

### Functional data can outperform computational predictors in pathogenicity classification

Computational predictors, such as AlphaMissense (*48*), have been increasingly used to infer variant pathogenicity, guiding both genetic interpretation (*49*–*52*) and early-stage drug discovery efforts (*53*). By leveraging evolutionary conservation, structural information, and previously-assigned pathogenicity labels, these models aim to classify missense variants as benign or pathogenic, often serving as a filter for identifying disease-associated alleles and for prioritizing targets for therapeutic intervention. To assess how well AlphaMissense predicts pathogenicity for *RHO* variants, we compared the AlphaMissense predictions to the known ClinVar clinical significance category. Of the seven likely benign or benign *RHO* missense variants, AlphaMissense predicted one as ambiguous and another as likely pathogenic (Fig. 5B), in contrast to our trafficking score, which was normal for all benign variants (Fig. 5A). Next, we compared our trafficking scores with the population allele frequencies reported in gnomAD and observed that high-frequency variants uniformly score in the normal range of the trafficking assay (Fig. 5C). When allele frequency data were compared to AlphaMissense, a larger proportion of high frequency alleles (61.2%) and low frequency alleles (82.7%) are predicted to be likely pathogenic (Fig. 5D) suggesting that AlphaMissense has low specificity (“over-calls”) when predicting pathogenicity. Compared to our composite trafficking score, AlphaMissense undercalls mistrafficking variants in the unstructured N-terminus and likely inaccurately predicts very high pathogenicity rates for the majority of uncharged transmembrane variants and tryptophan and cysteine substitutions (Fig. 5E). Note that the color scale for Fig. 5E was set to reflect the assigned numerical meaning of the AlphaMissense pathogenicity categories. Neither the trafficking score nor AlphaMissense was able to correctly predict pathogenicity for Class 1 variants in the C-terminal tail.

We evaluated 53 other computational metrics as well, demonstrating that the trafficking score provides distinct information not captured by the other known computational predictors (fig. S4). These results highlight that existing in silico tools can exhibit biases in pathogenicity predictions, can fail to offer the mechanistic detail necessary for guiding precision drug development, and would benefit from the integration of functional data into multimodal training datasets to improve accuracy.

### Broad correction of variant trafficking defects by a non-retinoid pharmacological chaperone

Protein misfolding diseases, where protein variants fail to fold and do not localize to the proper subcellular compartment, represent a substantial therapeutic challenge. Pharmacological chaperones, or ‘correctors’, offer a promising therapeutic approach, serving to stabilize misfolded proteins and promote their release from the ER and/or proper trafficking. Successful corrector therapies, such as ivacaftor for *CFTR* in cystic fibrosis (*54*) and migalastat for *GLA* in Fabry disease (*55*), have demonstrated the clinical potential of this strategy. *RHO*-adRP is particularly well-suited for a corrector therapy, as many pathogenic RHO variants lead to protein misfolding and retention in the ER. Previous work has explored retinoid analogs of the natural RHO ligand, 11-*cis*-retinal, which have shown low levels of corrector activity for variants like P23H (*56*–*58*), but are light-sensitive and covalently conjugate to RHO. One study identified a non-retinoid pharmacological chaperone, YC-001, that reversibly binds the same orthosteric site for enhanced and more potent restoration of folding for four Class 2 variants with baseline trafficking defects: P23H, D190N, G106R, and P267L (*31*). Another study used virtual screening to identify two higher-affinity non-retinoid ligands (JC3 and JC4) that increase cell-surface expression across 36 of 123 variants tested (*59*). The observed “chaperone-mediated” increase in surface trafficking observed with YC-001 and similar molecules could reflect a combination of underlying mechanisms, including improved folding, secretory trafficking, and/or stabilization of receptors at the plasma membrane by reducing their turnover or degradation (*60*).

**Fig. 6.**
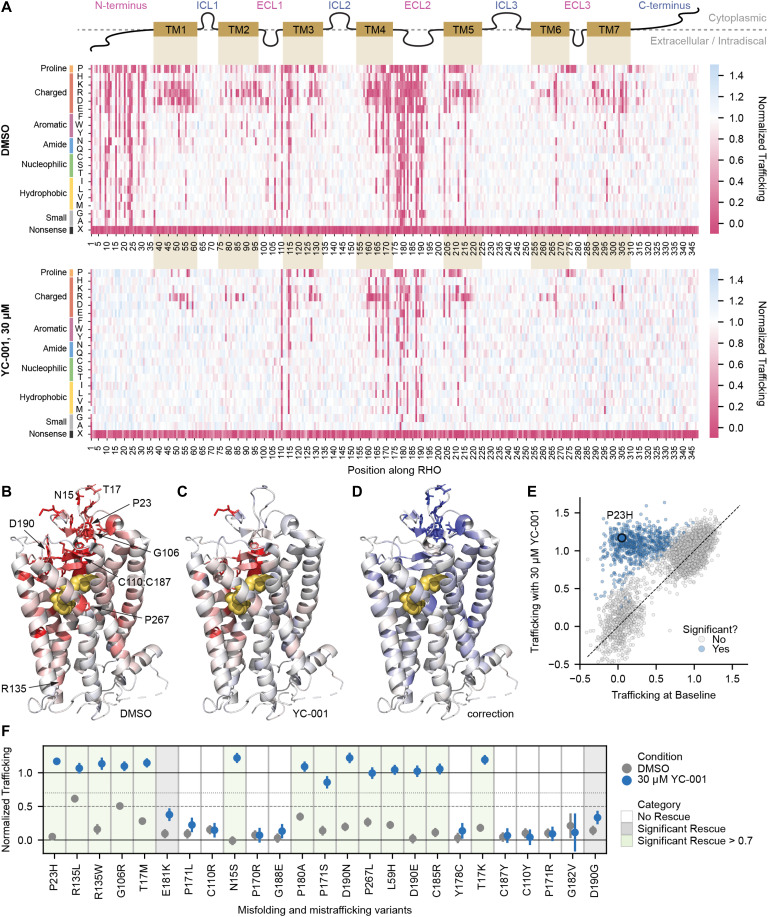
YC-001 broadly rescues trafficking for a majority of misfolding and mistrafficking variants. (**A**) Heatmaps showing surface trafficking for Method 2 at baseline with the DMSO vehicle (top; same as Fig. 2D bottom) and with 30 μM YC-001 treatment (bottom) for all missense and nonsense variants. Heatmaps are normalized and colored as in Fig. 2. (**B** to **C**) RHO structure (PDB: 1F88) colored according to the average trafficking score at each position with (B) DMSO vehicle or (C) 30 μM YC-001 (red: greater average trafficking defect). Side chains are shown for residues with an average trafficking score <0.5. (**D**) RHO structure is colored according to the degree of correction, defined as the difference in average trafficking scores at each position between 30 μM YC-001 and DMSO vehicle (blue: greater correction). (**E**) Scatterplot comparing trafficking scores at baseline with the DMSO vehicle (*x*-axis) versus with 30 μM YC-001 (y-axis). Variants with significant differences between treatment conditions (5% FDR) are shown in blue, with 550 variants above the y = x line (significantly increased trafficking with YC-001) and none below. (**F**) Rescue plots showing surface trafficking with vehicle (gray) and with 30 μM YC-001 (blue) for higher-frequency misfolding and mistrafficking variants identified in clinical cohorts, ordered by relative frequency, with mean and 95% confidence intervals. Thresholds for moderate confidence (<0.7) and high confidence (<0.5) trafficking defects are indicated as dotted and dashed lines, respectively. Variants that show no rescue have a white background. Variants that show significant rescue (5% FDR) to a level below 0.7 have a gray background. Variants that show significant rescue to a level above 0.7 have a green background.

To investigate the corrective potential of YC-001 more broadly across mistrafficking variants, we performed the Method 2 DMS assay in the presence of 30 μM YC-001, the approximate EC_90_ concentration for P23H trafficking rescue (*31*). Of the 913 moderate and high confidence mistrafficking missense variants identified by Method 2 in the absence of corrector drug, 550 missense variants (60%) showed significant rescue at a 5% FDR after 24 hours treatment with 30 μM YC-001 (Fig. 6, movie S1). This included most N-terminal and many transmembrane and ECL2 mistrafficking variants (Fig. 6, B to D). Variants not amenable to correction included those at C110 and C187, which form a disulfide bridge critical for folding and functioning of rhodopsin (*33*), as well as other residues within ECL2 that may be important for coordinating protein-drug interactions (Fig. 6, A and F). Among the most common misfolding and mistrafficking variants identified in clinical cohorts in the United States, the majority of variants exhibited significant increases in trafficking with YC-001 treatment (Fig. 6F), suggesting a substantial fraction of *RHO*-adRP cases caused by misfolding and mistrafficking variants may be addressable by small molecule correctors.

## DISCUSSION

In this study, we comprehensively mapped the functional impact of every possible missense variant in *RHO* using two complementary deep mutational scanning (DMS) assays in HEK293T cells, quantifying the effects on folding and surface trafficking as well as responsiveness to a prototype non-retinoid corrector. This work more than tripled the number of variants with experimentally defined pathogenic trafficking defects relative to prior decades of work and showed that a majority of mistrafficking variants are functionally rescuable. Beyond *RHO*, this study illustrates how multiplexed functional genomics can connect genotype to mechanism to therapeutic potential, at scale, offering a roadmap for genetics-informed drug development.

Traditional variant interpretation distinguishes pathogenic from benign alleles but does not specify how disease arises or whether the variant is pharmacologically addressable. Here, we shift toward an integrated genotype-function-therapy framework: we first distinguish between disease mechanisms and then between “correction-responsive” and “correction-refractory” variants. In this view, variant classes carry prognostic and therapeutic implications, informing diagnosis, counseling, and genotype-guided trial design.

Results from our flow cytometry immunoassay and proximity-based transcriptional reporter assay strongly cross-validated each other, additionally aligning with prior lower-throughput studies (*28*–*30*, *61*). The assays reproducibly measured surface trafficking defects for most annotated Class 2 variants (*10*, *28*), which result in misfolding and ER retention (*62*), and found no defects for Classes 1, 5, 6, or 7, which cause disease by other mechanisms (*10*, *62*). The limited differences between methods were mechanistically informative: substitutions within the antibody epitope can reduce immunostaining without lowering true surface abundance, while Class 3 variants that accelerate endocytosis (e.g., at the conserved arginine 135 in the E/DRY motif) (*41*, *63*) can depress immunostaining relative to the proximity sensor, which is less sensitive to plasma membrane residence time. Overall, such divergence likely reflects kinetics or compartmentalization rather than measurement error, highlighting the value of complementary readouts for mechanistic resolution.

The resulting landscape clarifies structural constraints in RHO biology (*64*, *65*): pronounced intolerance in the extracellular/intradiscal N-terminus and ECL2 “beta-plug” (*66*), sensitivity at disulfide-forming cysteines, and susceptibility of transmembrane helices to charged and proline substitutions (*67*–*69*). While RHO shares constrained motifs such as the (Wxx)GxxxC “structural hinge” with other Class A GPCRs (*67*), the pronounced sensitivity of the beta-plug region appears RHO-specific. Opsins likely bind retinal through transient interhelical pores rather than the more “open” extracellular domains present in most GPCRs, enabling the extracellular/intradiscal region to evolve roles in thermal stability and activation (*10*, *70*). These features may be exploitable for tailored drug design.

Functionally, the dominant disease mechanism captured here is misfolding and ER retention that elicits ER stress and UPR activation. This view reconciled edge cases (e.g., ER retention for R135W (*63*, *71*, *72*); dual Class 2/Class 4 features for T17M (*10*)), reinforcing misfolding as the predominant actionable mechanism in *RHO*-adRP. Variants whose primary dysfunction does not reduce surface trafficking (Classes 1, 5, 6, 7) showed no ER stress signature in this system. The framework established here can extend beyond trafficking to interrogate a broader spectrum of RHO phenotypes, including polarized outer-segment trafficking (Class 1), G-protein coupling (Class 5), arrestin recruitment/internalization (Class 6), and dimerization/complex assembly (Class 7).

Composite trafficking scores displayed a bimodal distribution, enabling pragmatic thresholds for pathogenic trafficking levels. Conservative cutoffs provide high specificity, with benign/likely benign missense variants clustered at high scores; however, sensitivity is inherently imperfect for mechanisms that do not affect trafficking. When the statistical power for predicting pathogenicity of the composite trafficking score is calculated using an OddsPath value, a binary assay cutoff of 0.7 produces OddsPath values that represent “strong” evidence for pathogenic (PS3) or benign (BS3) classifications. These calculations are based on ClinVar and population data as the gold standard; however, clinical testing pipelines inherently underreport benign variants to ClinVar, creating an ascertainment bias that affects the calculations (*73*). In this context, the strong BS3 rating should be used with caution. Ideally, a probabilistic framework that incorporates replicate variance, positional context, ER-stress readouts, and clinical priors could further improve calibration.

Results from this study also clarify discrepancies in the literature. A small subset of previously labeled “Class 2” variants with normal trafficking in our system and similar (*28*) systems may reflect sequence differences in earlier studies (e.g., bovine backbone) (*74*); cell type differences in protein degradation or posttranslational modification (*75*); differences in folding, maturation, and trafficking through the secretory pathway (*76*) (though these are thought to be universal (*77*)); or genuine non-trafficking mechanisms. For example, G51V, T58M/R, L125R, A164V, and C167R had completely normal trafficking scores in this study despite being identified as Class 2 variants in a prior review (*10*). G51V was originally described as accumulating normally in the plasma membrane of 293S cells (*78*) and impairing signaling without gross misfolding (*79*) but was later misclassified as ER retained/Class 2 based on biochemical data (*10*, *80*). For C167R, localization was originally reported to be abnormal in SF9 insect cells (*81*), but more recent studies suggest less severe trafficking impairment than expected for a canonical Class 2 variant (*28*, *58*). More systematic recent trafficking/localization studies in mammalian cells are broadly consistent with our results (*29*, *58*), and therefore the these outliers likely reflect historical classification differences rather than assay failure per se. Overall, the implementation of a comprehensive assay in this study allows for comparison to and consolidation of older classifications from the literature.

A central result is that most variants with trafficking defects are significantly corrected by the non-retinoid corrector, YC-001. The resulting “responder classes”—sets of variants sharing both mechanism and pharmacologic rescue—create a direct bridge from functional genomics to therapeutic development. In RHO, responder enrichment in the N-terminal/ECL2 regions contrasts with refractory behavior at cysteines forming the intradiscal disulfide bond (*82*) and residues likely involved in binding YC-001. These patterns, together with the tool-compound profile of YC-001, support data-driven medicinal chemistry hypotheses to increase potency, retinal exposure, and activity against refractory variants.

In parallel, a companion study (*83*) leveraged the Method 2 trafficking map (plus a single-plex assay for non-missense variants) to categorize patient variants and relate functional scores to clinical outcomes, underscoring translational utility. Functionally grounded evidence can help reclassify variants of uncertain significance, improving diagnostic yield and counseling, and define molecular subsets for variant-stratified or response-enriched trials, thereby improving power in rare-disease settings.

These experiments define a strong foundation while also highlighting avenues for future work. Our assays were conducted in HEK293T cells and focus on surface trafficking, and as such do not fully recapitulate photoreceptor biology in ciliated polarized cells, despite core features of protein misfolding and ER quality control being conserved. Corrector effects were assessed with a single prototype compound and under a limited set of conditions, likely underestimating the full landscape of pharmacologic responsiveness. These points motivate extensions to photoreceptor-like cell systems, broader mechanistic coverage, and comparative testing of multiple correctors. More generally, integrating multi-dimensional DMS readouts with transcriptomic, proteomic, and structural datasets could enable richer mechanistic models of pathogenicity and drug response. Such empirical, high-resolution data also provide a powerful substrate for next-generation machine learning, enabling retraining of pathogenicity predictors and transfer learning across gene families and disease contexts.

By linking variant-level dysfunction to correction-responsiveness, this work reframes how functional genomics informs therapy. In *RHO*, a coherent picture emerges: misfolding is the predominant actionable mechanism; many such variants are correctable; and responder classes can be prospectively identified. The same logic is portable to other GPCRs with folding/trafficking defects, to ion channels and transporters in Mendelian disease, and to oncology contexts where variant-specific mechanisms dictate pharmacology. Extensions of this approach, such as using DMS for high-throughput generation of dose response curves or comparative analysis of residues rescued by different compounds, can support rational drug design, patient selection, dose optimization, and clinical trial designs. As multiplexed functional assays are increasingly coupled to clinical genomics, we anticipate a virtuous cycle in which empirical maps refine diagnostics, enable efficient variant-informed trials, and accelerate drug discovery, positioning DMS as a linchpin of genetics-informed drug development.

## MATERIALS AND METHODS

### Cell culture for Method 1

For Method 1, HEK 293T cells (ATCC, CRL-3216) were maintained in Dulbecco’s modified Eagle’s medium (DMEM) (Thermo Fisher, 11–995-065) supplemented with 10% fetal bovine serum ( Hyclone, Cat.SH3007103). The cells were maintained at 37°C in 5% CO_2_.

### Vectors and library preparation for Method 1

For the design of the saturation mutagenesis variant library, each amino acid position (a.a. 2–348) in the reference sequence of RHO was systematically altered to 19 different amino acids, as well as synonymous and stop codons that serve as assay controls. The wildtype human RHO sequence was based on the open reading frame (ORF) in NM_000539.3. Variant codons (usually 1 per amino acid position) were selected to favor 2- or 3- nucleotide changes rather than 1-nucleotide changes when possible, to avoid collisions with sequencing errors. Variant codons which would create restriction sites used in the cloning reaction were excluded as well.

For synthesis of this library (Site Saturation Variant library product; Twist Biosciences), an error-free wild-type Rhodopsin template was synthesized as the starting point for all variants in the library. This WT template DNA was divided into aliquots and placed in individual wells of 96-well plates. To each well, outer primers were added, flanking the specific region of the gene where the variant would occur, enabling site-directed mutagenesis. PCRs were performed using high-fidelity polymerase to ensure the variant was incorporated into the newly synthesized DNA according to manufacturer’s instructions (Twist Bioscience). After PCR, the DNA from each well was normalized to ensure equal representation of all variants in the final library. The DNA from all wells was pooled together, and quality control (QC) was performed by NGS to verify the presence and accuracy of the desired variants. The ORF was flanked with adapters suitable for restriction digestion and ligation into NheI and BamHI sites of pMT_025 vector (Addgene #158579). The pMT_025 was provided by the Genetic Perturbation Platform, Broad Institute of MIT and Harvard and contains two components: 1) an EF-1α promoter that drives the rhodopsin ORF and 2) an SV40 promoter that drives puromycin resistance. The fragments were ligated into the NheI and BamHI restriction site of pMT_025 vector using T7 DNA ligase. Post clean-up, the products were transformed, and the plasmid DNA library was isolated using the Maxi-prep kit (Qiagen HiSpeed Maxi, 12663) and subjected to NGS. The mutagenesis library was characterized to identify and distinguish intended variants from variants arising from errors during library synthesis. To assess clone distribution, we scaled raw variant counts in the plasmid library by their local read coverage, and the raw counts were adjusted and scaled to account for uneven sequencing depth. The scaled values were log-transformed for better visualization, and the resulting values were log_2_-transformed to form the histogram bins. Briefly, the steps involved trimming the flanking sequences, aligning overlapping paired-end reads, applying quality and length filters, and identifying and quantifying sequence variation and the abundance of each variant. The variant abundance distribution revealed a representation of >99% of the intended substitutions in the variant pool.

### Lentivirus production for Method 1

HEK293T packaging cells were cultured in DMEM (Thermo Fisher, 11965092) supplemented with 10% FBS (Hyclone, SH30071.03). Briefly, the cells were transfected with the plasmid DNA lentiviral library, psPAX2 (Addgene #12260), and pMD2.G (Addgene #12259) using TransIT-LT1 transfection reagent (Mirus Bio, MIR 2304). Details of the protocol can be accessed at http://www.broadinstitute.org/rnai/public/resources/protocols. Media was replaced and viral supernatant was harvested at 24 and 48 hours. Serial dilutions of a standard and library were used to determine the viral titer in A549 cells. The transduced cells were selected using puromycin (1.5 μg/mL, Gibco, A1113803) for two days. The AlamarBlue assay was performed, and the standard curve method was used to determine the linear range, sensitivity of the assay, and the viral titer of the *RHO* library.

For library transduction, HEK293T cells were cultured and expanded in T225 flasks. Upon reaching 80% confluency (∼45 million cells), the cells were washed with PBS and dissociated using 0.25% trypsin. To ensure adequate coverage/representation of the *RHO* library, each variant was transduced into a minimum of 500 cells. Additionally, the cells were transduced at a low multiplicity of infection (MOI = 0.3) to ensure single-copy transgene integration. This ensures the required coverage/representation of the library containing up to 7,299 variants, with an estimate of 500 transduced cells per variant. With a target cell transduction efficiency of <30%, a total of 14.7 million cells were transduced by combining 48.9 mL of viral supernatant (titer of 2.42 × 10^5^) and polybrene (8 μg/mL, Sigma, H9268) and plated 2 mL per well into a total of four 6-well plates. Subsequently, the plates were sealed and centrifuged at 2000 g for 50 minutes and maintained at 37°C with 5% CO_2_ overnight. The following day the media was replaced with fresh media. After 48 hours, the cells were dissociated using 0.25% trypsin, pooled and re-seeded at a density of 5 × 10^6^ cells per T225 flask in 50 mL growth media (a total of ten flasks). Selection pressure was applied by adding puromycin at a final concentration of 800 ng/mL. On day 5–6, transduction efficiency was estimated in this fraction by immunostaining followed by MACS quant flow cytometry. To determine the infection efficiency, 3.5 × 10^5^ transduced and non-transduced cells were plated with and without puromycin (800 ng/mL). The transduction efficiency was estimated as the ratio of average cell count of puromycin^+^ cells/average cell count of puromycin^−^ cells.

### Immunostaining and FACS for Method 1

Cells were washed with 10 mL DPBS per T225 flask and dissociated using 0.25% trypsin. The flasks were gently tapped and as soon as the cells detached, 10 ml complete medium was added to quench the reaction. The cells from all flasks were pooled, centrifuged at 1000 rpm for 5 minutes at room temperature. The cell pellet was washed with 10 mL of DPBS and centrifuged at 1000 rpm for 5 minutes. The pellet was resuspended and fixed in 10 mL (10 million cells/mL) 4% paraformaldehyde (Electron Microscopy Sciences, 15710) in PBS for 20 minutes at room temperature. After PBS wash, blocking was performed with 3% BSA/PBS for 10 minutes followed by incubation in RetP1 RHO primary antibody (Sigma, O4886, 1:2000 dilution) for 30 minutes at room temperature. The cells were collected by centrifugation at 1200 rpm for 5 minutes, washed with 10 mL PBS, followed by incubation with anti-mouse 488 secondary antibody (Thermo, A21121) for 30 minutes in the dark at room temperature. After incubation, the cells were washed with PBS and resuspended at a density of 1 × 10^7^ cells/mL in PBS. All the cells were passed through a 40 μm cell strainer (Corning, 352340) before acquisition and sorting using a Sony cell sorter machine (SH800 or MA900). The sorting utilized a 100-micron nozzle, with the sort mode set to purity. The flow-rate was maintained between 2500–3000 events/sec, and sample pressure was kept constant at 7 psi, and was held at 4°C. After confirming proper staining using positive and negative controls, cells were sorted from RHO^high^ and RHO^low^ expressing bins. The top (75–100%) and bottom (0–25%) quartiles of fluorescent intensity cell were sorted and collected separately as RHO^high^ and RHO^low^ samples, respectively. Using the MA900 sorter, 8.5 × 10^6^ cells RHO^low^ cells and 12.3 × 10^6^cells RHO^high^ cells were sorted. Using SH800 sorter, 4.5 × 10^6^ cells RHO^low^ cells and 6.6 × 10^6^ cells RHO^high^ cells were sorted. The sorted cells were pelleted down and subjected to DNA extraction.

### Extraction of DNA from FACS sorted cells for Method 1

The recommended reagent volume described below is for the extraction of genomic DNA from 1 × 10^6^ fixed cells. The volume was scaled up based on the number of cells sorted. For Method 1, the cells were lysed overnight, in 200 μL Quick-extract buffer (Lucigen, NC0302740) supplemented with 3.75 μL proteinase K (Thermo, EO0491), at 60°C, with 600 rpm shaking. Post lysis, 50 μL of 5 M NaCl (Thermo Fisher, AM9760G) was added and mixed thoroughly by inverting the tube 50 times and incubated on ice for 10 minutes. Following the incubation, 3.5 μL of RNAase A (Thermo, EN0531) was added and mixed thoroughly by inverting the tube 50 times and incubated for 10 minutes at room temperature. The samples were centrifuged at 12,000 rpm for 10 minutes at room temperature. The supernatant was carefully transferred into a fresh tube and mixed with equal volume of Isopropanol and mixed thoroughly by inverting 20 times and incubated for 10 minutes at room temperature. The tubes were left undisturbed, which allowed the phases to separate. The top phase was carefully aspirated and discarded. Equal volume of monarch DNA binding buffer (Monarch Kit, T1030S) was added and the samples were mixed thoroughly by inverting the tube 10–15 times and transferred into the Monarch columns. The columns were centrifuged at room temperature for 1 minute, discarding the flowthrough. The columns were washed with 500 μL DNA Wash Buffer and centrifuged at max speed for 1 minute. The columns were then transferred into a clean 1.5 mL microfuge tube. Elution buffer was added (30 μL) to the center of the matrix, incubated for 1 minute, centrifuged at max speed for 1 minute to elute DNA. The concentration was measured using Nanodrop spectrophotometry and integrity was confirmed by electrophoresis by running 1 μg DNA on a 1% TAE agarose gel.

### Assay deconvolution for Method 1

The rhodopsin ORF from the genomic DNA was PCR amplified using primers: Forward: 5′ ATTCTCCTTGGAATTTGCCCTT−3′, Reverse: 5′- CATAGCGTAAAAGGAGCAACA −3′. PCR primers were designed to have ∼100 bp flanking sequences on either side of the ORF region and was performed using Q5 Hot Start High-Fidelity 2X Master Mix (NEB, M0494S) using the program: 95°C for 30 seconds, 98°C for 10 seconds, 69°C for 30 seconds, 72°C for 2.5 minutes (35 cycles) and 72°C 2 minutes and 4°C hold. A full 96-well PCR plate was used for each set, and each well with a reaction mix of 50 μL and 250 ng of gDNA. Post-PCR, the products were separated on 1% agarose, extracted and purified first using QIAquick Gel Extraction Kit (Qiagen, 28704) followed by AMPure XP kit (Beckman Coulter, A63881). PCR products from each set were subjected to transposon-mediated fragmentation, indexed and sequenced on an Illumina next-generation sequencing platform. Screen deconvolution was performed with Analyze Saturation Mutagenesis (ASM) software, as described (*84*). Briefly, paired-end reads were aligned to the reference sequence, filtered, trimmed, and variant counts were calculated and the output files were parsed, annotated, and counts were merged into a single .csv file.

### Statistical Analysis for Method 1

For both datasets, variant counts were modeled using the *glmmTMB* package with a negative binomial linking function, using R (version 4.4.1). For Method 1, a mixed-effects model was fitted to each position to estimate the effect sizes of amino acid, gate conditions, and amino acid and gate interactions, while accounting for replication effects. Estimated marginal means were calculated, and linear contrasts were performed as the final estimate of relative abundance, which is the difference between abundance in high and low gates. The results were min-max scaled to read depth, with the mean linear contrast estimates of wild type as the maximum and the mean linear contrast of nonsense variants as the minimum and this resulted in most relative abundance values falling between 0 and 1.

### Variant library design and oligonucleotide pool synthesis for Method 2

The UniProt protein sequence for the 348 amino acid (aa)-long human rhodopsin protein (P08100) was used as the reference for library design and codon optimized at the DNA level for ease of DNA synthesis and downstream molecular cloning. All single-residue missense and nonsense variants (6,960 in total at the protein level) were designed in silico using up to three of the most common codons where possible (https://github.com/octantbio/rho-dms). Only nucleotides within the codon of interest were changed except in cases where the substitution introduced a homopolymeric sequence that could interfere with DNA synthesis or a restriction site that could inhibit proper molecular cloning. The 348 amino acid (aa)-long sequence was divided into five chunks between 68 to 70 aa in length to be within current pooled oligonucleotide synthesis length limits (≤300 nt) after the addition of sequences required for amplification and cloning. Oligonucleotides for all five chunks were ordered in a single oligonucleotide pool from Twist Bioscience, with each chunk containing unique flanking sequences. Upon arrival, the lyophilized oligonucleotide pool was resuspended in IDTE pH 8.0 (IDT, 11–05–01-09) to 10 ng/μL, aliquoted, and stored for long term storage at −80°C.

### Step 1 library cloning for Method 2

Each chunk was amplified in two rounds of PCR using NEBNext Ultra II Q5 Master Mix (NEB, M0544X), 0.2 ng/μL oligonucleotide pool template, and 5 μM forward and reverse chunk-specific primers. The first PCR selectively amplified the chunk of interest from the oligonucleotide pool, while the second PCR appended a variable 21-nucleotide barcode sequence and constant region for downstream cloning steps to each chunk sublibrary (cycle numbers for amplification determined empirically by qPCR).

Chunk-specific base cloning vectors were designed to contain a pair of SapI restriction upstream of a constant portion of the *RHO* gene. Golden Gate cloning with SapI (NEB, R0569L), high concentration T4 DNA Ligase (NEB, M0202M), 0.5 nM of pre-digested vector, and 0.5 nM amplified insert was performed in T4 DNA Ligase Reaction Buffer (NEB, B0202S) to insert the barcoded variable regions into the appropriate base cloning vector using the following cycling conditions: 37°C × 5 min initial digestion, 65 cycles of 37°C × 5 min digestion followed by 16°C × 5 min ligation, 37°C × 10 min final digestion, 70°C × 20 min denaturation, and 10°C hold. Golden Gate reaction products were concentrated using the DNA Clean & Concentrator-5 kit (Zymo Research, D4004) and eluting in 6 μL fresh MilliQ water. Two microliters of this concentrated Golden Gate product were electroporated into 50 μL of Endura Electrocompetent Cells (Lucigen, 60242–2) in Gene Pulser/MicroPulser Electroporation Cuvettes with a 0.1 cm gap width (Bio-Rad, 1652089) using a Bio-Rad MicroPulser Electroporator (Bio-Rad, 1652100) on program “Ec.1” with voltage 1.8 kV and time constants between 5.5–5.7 ms. Immediately following electroporation, 2 mL of pre-warmed Recovery Media (Lucigen, 80026–1) were added to the cuvette to collect the electroporated bacteria, which were transferred to a 14 mL round-bottom tube and shaken at 200 rpm at 37°C × 1 hr. to allow recovery and expression of the plasmid-encoded kanamycin resistance gene. A portion of the recovery culture was serially diluted and plated on LB agar plates with 50 μg/mL kanamycin sulfate (Teknova, L1025) for estimation of electroporation efficiency and library diversity. The remaining recovery culture was inoculated into 50 mL 2XYT Broth (Teknova, Y2140) with kanamycin and grown for 16 hours at 30°C. For electroporation reactions yielding greater than 1e6 colony forming units, plasmid libraries were purified from the liquid cultures using the ZymoPURE II Plasmid Purification Midiprep Kit (Zymo Research, D4201) according to the manufacturer’s instructions.

### Barcode-variant mapping for Method 2

Following Step 1 library cloning, each plasmid contained a designed variable chunk sequence adjacent to an unknown random barcode and separated by a pair of Esp3I restriction sites. To map the relationships between the barcodes and variable chunk sequences, this region on the plasmid libraries was amplified by PCR using the NEBNext Ultra II Q5 Master Mix and 5 μM forward and reverse primers containing sequences for grafting to an Illumina flow cell (single indexing). Two technical replicate amplicon sequencing libraries were prepared for each chunk and were subjected to 2x150 paired-end sequencing using the 300-cycle NextSeq 2000 P3 Reagent kit (Illumina, 20040561) on an Illumina NextSeq 2000 instrument. Resulting raw BCL files were demultiplexed with bcl2fastq and processed as described in (*85*). Briefly, barcode and oligo sequences were extracted, mapped against a reference of all designed RHO sequences, counted, and filtered. Each oligo-barcode pair was required to: (i) be the appropriate length, (ii) have ≥ 3 supporting read counts, (iii) have a purity ≥ 0.75 (defined as the fraction of all reads from the barcode which support the pair). Each library chunk was mapped separately, and then all resulting chunk maps were concatenated to generate the final barcode oligo map. Across the 12 assay samples, the median number of quantified barcodes per variant was 57.5.

### Step 2 library cloning for Method 2

A second Golden Gate cloning step was performed to insert the remaining elements (regulatory elements and upstream constant portion of the rhodopsin sequence) into the Esp3I restriction sites between the barcode and variable chunk sequence. The elements to insert for each chunk were encoded in chunk-specific donor vectors, and both these donor vectors and the plasmid libraries from Step 1 cloning were pre-digested with Esp3I (NEB, R0734L) prior to their use in Golden Gate reactions with the BsmBI-v2 NEBridge Golden Gate Assembly Kit (NEB, E1602L; note, BsmBI is an isoschizomer of Esp3I) and T4 DNA Ligase Reaction Buffer. Cycling conditions for these reactions were as follows: 42°C × 5 min initial digestion, 65 cycles of 42°C × 5 min digestion followed by 16°C × 5 min ligation, 42°C × 15 min final digestion, 70°C × 20 min heat inactivation, and 10°C hold. Golden Gate reactions were concentrated, Endura Electrocompetent Cells were electroporated, and plasmid libraries were purified as described in the Step 1 library cloning Methods subsection. At this step, each plasmid contains the complete rhodopsin variant expression and barcoded trafficking reporter cassettes, with one barcode-variant combination per plasmid.

### Mammalian cell engineering for Method 2

All cell engineering was performed with a clonal HEK293T cell line constitutively expressing the reverse tetracycline transactivator (rtTA) and containing a Bxb1 recombinase-based landing pad in one copy of the *H11* safe harbor locus. This line was cultured in Gibco DMEM with High Glucose and GlutaMAX Supplement (Thermo Fisher Scientific, 10566024), hereafter DMEM, supplemented with 10% qualified FBS (Thermo Fisher Scientific, 26140079). TrypLE Select Enzyme without phenol red (Thermo Fisher Scientific, 12563029) was used for cell dissociation during routine passaging.

For each of the five final plasmid libraries, 5.5e6 cells were seeded into each of ten 10-cm dishes in 12 mL of DMEM +10% qualified FBS. The following day, cells were co-transfected with 11.8 μg of the appropriate variant plasmid library and 2.92 μg of the Bxb1 recombinase expression plasmid using 29.4 μL of P3000 reagent and 44.2 μL of Lipofectamine 3000 reagent (Thermo Fisher Scientific, L3000075) diluted in Opti-MEM (Thermo Fisher Scientific, 31985088), using incubation times as indicated in the manufacturer’s protocol, for site-specific, single-copy integration of one barcode-variant combination per cell. Three days later, the ten 10-cm dishes were expanded to ten 15-cm dishes without discarding any cells, and, the next day, puromycin (Thermo Scientific, A1113802) was added at 1 μg/mL to each dish to cull cells that did not properly integrate the *RHO* variant expression/reporter cassette. A full media change with fresh 1 μg/mL puromycin was performed three days later. Four days later, cells were dissociated and counted, and 30e6 cells were seeded in 40 mL media into each of four T225 flasks. Three days later, cells were dissociated, counted, and prepared for cryopreservation in BAMBANKER (VWR, 101974–112).

### Assay for Method 2

Individual chunk cell libraries were thawed, allowed to expand, counted, pooled in equal amounts, and plated at 17e6 cells per 15-cm dish, with four replicate dishes per condition. One day later, cells were treated with 1) 2 μM doxycycline hyclate (ApexBio, A4052) to induce rhodopsin variant expression and 2) either DMSO or the YC-001 corrector molecule (MedChemExpress, HY-124717). Approximately 18 hours later, media was aspirated and cells were lysed using 4 mL ice-cold Buffer RLT (Qiagen, 79216) containing beta-mercaptoethanol (Sigma Aldrich, M3148-25ML) for RNase inhibition. Dishes were scraped using cell lifters (Bio Basic, SP91151), lysates were transferred to 5 mL tubes, and lysates were homogenized by repeated aspiration and dispensing using 10 mL syringes with 18-gauge blunt tip needles (VWR, NB18212). Total RNA was purified from 350 μL of the homogenized lysate using the RNeasy Plus Mini Kit (Qiagen, 74134) column-based clean-up that included on-column treatment with DNase (Qiagen, 79254). RNA was eluted in 30 μL of the provided RNase-free water, and concentration and purity were assessed using a spectrophotometer.

cDNA containing reporter gene-encoded barcodes was selectively generated from the purified RNA using a reporter-specific primer and the SuperScript IV First-Strand Synthesis System (Thermo Fisher Scientific, 18091200) according to the manufacturer’s instructions. Reaction products were incubated with RNase A (Thermo Fisher Scientific, EN0531) and RNase H (provided in SuperScript kit) to digest away the original RNA template. NGS libraries were prepared for each sample with PCR using the NEBNext Ultra II Q5 Master Mix and 5 μM forward and reverse primers containing sequences for grafting to an Illumina flow cell (dual indexing). Amplicon sequencing libraries were subjected to 1x26 single-end sequencing using the 50-cycle NextSeq 2000 P3 Reagent kit (Illumina, 20046810) on an Illumina NextSeq 2000 instrument.

### Immunofluorescence staining and flow cytometry of rhodopsin truncating variants

HEK293T cells were maintained in DMEM supplemented with 10% FBS. Prior to the day of transfection, ∼1x10^5^ cells were seeded per well of a 12-well plate. For transfection, 2 μg of plasmids (WT *RHO*, W126X, G114D, G114X, F116X, F116X-(trunc:348 bp) were diluted in Opti-MEM (Thermo) and transfected using Lipofectamine 3000 reagent (Thermo, L3000015) following the manufacturer’s instructions. The following day, cells were dissociated using 0.25% trypsin, diluted and seeded on the chamber slides (Thermo Scientific, 12–565-843) coated with Laminin (Thermo, 23017015, 1:40 dilution in PBS for 1 hour at room temperature). The following day, the cells were washed and fixed with 4% paraformaldehyde (Electron Microscopy Sciences, 15710) and blocked with 3% BSA (Sigma-Aldrich). After blocking, the cells incubated with anti-rhodopsin antibody (Ret-P1 Sigma O4886, 1:2000 dilution) for 30 minutes at room temperature. After three washes with PBS, cells were incubated with Alexa Fluor anti-mouse secondary antibody (Thermo, A21121) for 30 minutes at room temperature. After three washes with PBS, the slides were layered and mounted using Prolong glass (Thermo, P36984). The slides were imaged under a fluorescence microscope (Eclipse Ti, Nikon). For flow cytometric analysis, the cells were dissociated and collected 48 hours post transfection, fixed and stained without permeabilization and analyzed using Sony MA900 (see immunostaining and FACS for Method 1).

### Statistical inference of variant effects for Method 2

For bioinformatic and statistical analysis, we applied a broadly similar approach as previously described (*85*). Briefly, raw BCL files were demultiplexed with bcl2fastq, and barcodes were extracted and counted from the resulting FASTQ files. Raw barcode counts from each sample were joined with the oligonucleotide-barcode map to generate the final count dataset. To infer variant effects across tested conditions, we applied a negative binomial generalized linear mixed model (NBGLMM). We fit one model per position, where each model included all barcodes derived from variants at that position as well as all wild-type barcodes from the chunk containing that position. The applied model is specified as

$${\text{count}}_{\text{ijkm}}∼\mathrm{NB}\left(\mu_{\mathrm{ij}},\theta \right)$$

$$\alpha_{k}∼N\left(0,\sigma^{2}\right)$$

$$\text{log}\left(\mu_{\mathrm{ij}}\right)=\beta_{i}^{\text{condition}}+\beta_{i}^{\text{variant}}+\alpha_{k}+\text{of fset}\left(\gamma_{m}\right)$$

For the ith condition, the jth variant, the kth barcode, and the mth sample. In this study, we consider two treatment conditions (DMSO and YC-001) and twenty possible variants at each position. Otherwise, we implement this model as previously described (*85*) including fitting with *glmmTMB* and coefficient and marginal mean computation with *emmeans*.

### Additional statistical testing

Variant-level effect estimates and standard errors from the negative binomial mixed-effects models (see above) were used for downstream inference. In all instances, 95% confidence intervals were defined as estimate ±1.96*(standard error) and used to summarize uncertainty. Variants were considered to have moderate or high confidence trafficking defects if the upper limit of the 95% confidence interval for variant trafficking was less than 0.7 or 0.5, respectively. To test whether YC-001 significantly altered trafficking for a given variant, we performed Wald tests on linear contrasts of the condition-specific log_2_ fold-changes (YC-001 versus DMSO). We computed Z-statistics and two-sided p-values using the contrast estimates and their standard errors, and adjusted for multiple testing across variants by computing false discovery rates using the Benjamini-Hochberg procedure. Variants with FDR < 0.05 were considered to have a significant change in trafficking and were classified as rescued or worsened (no instances) based on the sign of the contrast.

### Quantification of ER stress marker XBP1 mRNA in *RHO* variant cell lines by RT-qPCR

RHO variants were synthesized (39 in total, including WT) into doxycycline-inducible expression plasmids for piggybac transposase-mediated genomic integration. Each RHO variant plasmid was transfected alongside a piggybac transposase plasmid into HEK293T ADRB2−/− HEK293T cells, which had been transduced with lentivirus to overexpress rtTA-BFP. The plasmids were transfected using Lipofectamine 3000, then cultured in DMEM with 10% FBS. Puromycin (2 μg/mL) was administered to cells after 72 hours, and then cells were maintained in puromycin-containing media for 9 days to ensure all cells were fully selected. Two independent experiments for all RHO variants were carried out except variants V20L, P267T, N73K and W175L for which only a single experimental replicate was collected. RHO variant cell lines were plated onto Nunc MicroWell 384-Well tissue culture plates coated with collagen and poly-D-lysine, which were seeded at 20,000 cells per well in 30 μL of DMEM +10% FBS. To induce RHO transgene expression 2 μM doxycycline was administered to cells. After 24 hours media was spun out from plates, cells were washed with 1x PBS, then to extract RNA 20 μL of lysis reagent (NEB Luna Cell Ready lysis module, NEB E3032S) was added to each well. Plates were incubated for 10 min at room temperature, then lysate was mixed 3× and 15 μL was transferred to 384-well Enduro PCR plate and incubated as follows: 37°C × 15 min, 75°C × 15 min. RNA lysate was stored at −80°C until RT-qPCR. 1 μL of RNA template from each well was added to 9 μL of RT-qPCR master mix (Luna Universal Probe One-Step RTqPCR kit, NEB E3006E) containing three fluorogenic 5′-nuclease probes: XBP1s-FAM (Fwd: GCTGAGTCCGCAGCAGGT, Rev.: TGGGTCCAAGTTGTCCAGAATG, probe: 6-FAM/CCCATGGAT-ZEN-TCTGGCGGTATTGACT-IABkFQ), RHO-Cy5 (IDT PrimeTime Hs.PT.58.39668089.g) and GUSB-SUN (IDT PrimeTime Hs.PT.58v.27737538). For each probe forward and reverse PCR primers were added at 400 nM while each 5′ nuclease probe was added at 200 nM. RT-qPCR was carried out on QuantStudio 5 instrument (Applied Biosystems) as follows: 55°C × 10 min, 95°C × 1 min, followed by 45 cycles of (95°C × 10 sec, 60°C × 1 min), with fluorescent signal recorded after each cycle. Expression values were calculated using the ΔΔCt method (*86*). First, ΔCt values were calculated by normalizing XBP1s expression to the housekeeping gene GUSB for each sample. Then, ΔΔCt values were calculated by subtracting the mean ΔCt of the -dox (no doxycycline induction, representing baseline expression without RHO variant expression) control samples from the ΔCt of each variant sample within the same plate. Log_2_ fold changes were calculated as -ΔΔCt. To account for technical and biological variation across experiments (N = 1–2 independent replicates), a Bayesian mixed effects model was implemented using the *brms* R package (v2.22.0) (*87*). The model included variant as a fixed effect, with plate and plate:variant interaction as random effects. The model directly incorporated the standard errors of the measurements by modeling the variance. Estimates and 95% credible intervals were extracted for each variant. All log_2_ fold change estimates were normalized relative to wild-type RHO to facilitate comparison with composite trafficking data from the deep mutational scanning (DMS) experiments.

To assess the relationship between ER stress and trafficking defects, we performed a Bayesian meta-regression using *brms* with the composite trafficking data that accounted for measurement error in both variables:

log_2__normalized_ERstress|se(se_er_stress) ∼ me(trafficking_score, trafficking_SE)where brms::me() indicates measurement error modeling for the trafficking scores, and se() incorporates the standard errors of the ER stress estimates. Models were fit with 4 chains × 4000 iterations (2,000 warmup), adapt_delta = 0.95, yielding 8000 post-warmup draws with excellent convergence (R-hat = 1.00, ESS > 4000).

### Comparison to pathogenicity data and existing computational predictors

To obtain pathogenicity data from ClinVar for known variants, pathogenicity data for all single amino acid missense rhodopsin variants was downloaded February 5, 2025. ClinVar pathogenicity categories were re-coded into three levels: benign, VUS, and pathogenic. The “conflicting classifications” category was excluded. The pathogenicity data were linked to the prediction or trafficking data at the amino acid level. ClinVar ratings with at least “one star” evidence level were used. For computational predictors, pre-computed annotation was obtained for all single-nucleotide changes in the *RHO* gene from the dbNSFP4.9a. Only missense variants were analyzed, which excluded intronic and nonsense variants. After removing 5 sparsely populated predictors, rank scores for 53 predictors were included, ranging from 0 to 1. (Note that while the predictors are named “rankscore”, they are not homogeneously distributed between 0 and 1 like nonparametric ranks would be.) The combined trafficking score scale used in this study (0 = low trafficking, 1 = high trafficking) was reversed and scaled to match the predictors’ rank scores that had a range from 0–1 with 0 as benign and 1 as pathogenic, producing a trafficking_rankscore_homog. The trafficking scores and computational predictor scores were matched to the pathogenicity data at the amino acid level.

### Allele frequency and filtering criteria

Allele frequencies for all missense variants in *RHO* were obtained from gnomAD v4.1 and Regeneron Genetics Center Million Exome Variant Browser (accessed March 2025). A cutoff of 5 × 10^−5^ was established because any allele frequency above this level would be too high to be consistent with the prevalence of known *RHO*-associated RP in the population (e.g. estimating 1/4000 people with RP (*88*), 1/4 of RP is dominant (*11*), 1/3 of dominant RP is due to mutations in *RHO* (*88*), <1/2 fraction of any one new mutation within *RHO* mutations, which is 1/96,000 or 1 × 10^−5^). These criteria identified 55 relatively common missense variants used for fig. S3 and the OddsPath calculations. OddsPath calculations were performed. [See Brnich *et al.* (*24*) and this manuscript’s GitHub repository.] For these calculations, 140 missense variants were identified from ClinVar with likely pathogenic or pathogenic classifications. Comparisons between trafficking scores and the variants identified in databases were made at the amino acid level.
