# Supplementary material for: A comprehensive map of missense trafficking variants in rhodopsin and their response to pharmacologic correction
Source: Sci Adv. 2026 Jul 29;12(31):eaef3518. doi: 10.1126/sciadv.aef3518 (PMC13418928; doi:10.1126/sciadv.aef3518)
Supplement: Supplementary file 1 — Legends for data files S1 and S2 Legend for movie S1 Figs. S1 to S4 [file sciadv.aef3518_sm.pdf]

Science Advances
AAAS

Supplementary Materials for

A comprehensive map of missense trafficking variants in rhodopsin and their response to pharmacologic correction

Kannan V. Manian *et al.*

Corresponding author: Jason Comander, jason_comander@meei.harvard.edu

Sci. Adv. **12**, eaef3518 (2026)
DOI: 10.1126/sciadv.aef3518

The PDF file includes:

Legends for data files S1 and S2
Legend for movie S1
Figs. S1 to S4

Other Supplementary Material for this manuscript includes the following:

Data files S1 and S2
Movie S1

Other Supplementary Materials for this manuscript include the following:

Supplemental File 1. Trafficking score assay values, meta-analysis, and response to YC-001.

Supplemental File 2. RHO amino acid positions with domain annotation and average trafficking scores.

Supplemental Video 1. RHO structure (PDB: 1F88) colored according to the 25% percentile of the trafficking score at each position, with vehicle (left) and with YC-001 (right). The natural ligand, retinal, is indicated in yellow (for illustrative purposes only).

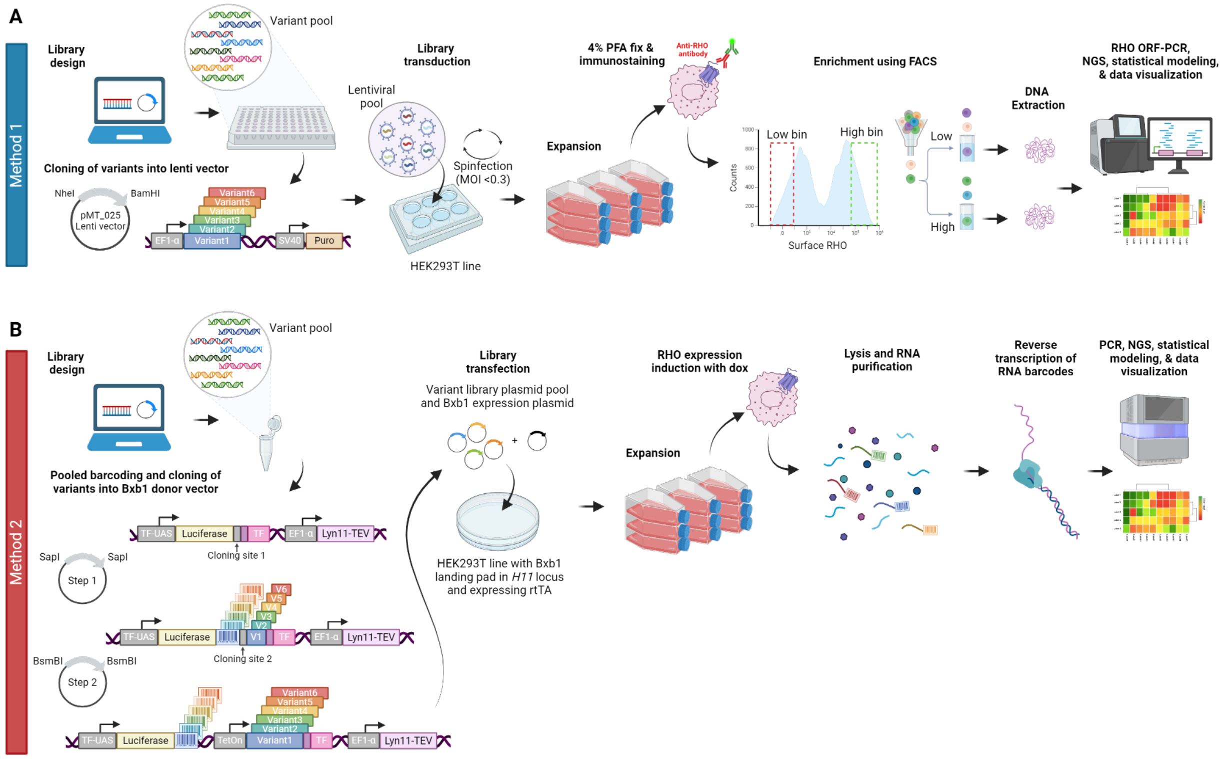

Figure S1. Detailed schematic of deep mutational scanning workflows. (A) Method 1 involves *in silico* library design, cloning variants into the pMT_025 lentiviral vector (NheI/BamHI = restriction sites, EF1α/SV40 = strong promoters, Puro = puromycin resistance gene), generating a lentiviral pool (MOI = multiplicity of infection), and transducing HEK293T cells at single copy. For the assay, cells are expanded, fixed with paraformaldehyde (PFA), and immunostained for surface-displayed RHO. The top and bottom quartiles are isolated by fluorescence-activated cell sorting (FACS) for downstream DNA extraction and library preparation (ORF-PCR = open-reading frame polymerase chain reaction). Libraries are subjected to next-generation sequencing (NGS), statistical modeling is performed on the sequencing counts to infer variant effects, and data are further analyzed and visualized. **(B)** Method 2 involves *in silico* library design, a multi-step variant barcoding and cloning process into a Bxb1 integrase-compatible donor vector, and transfecting this plasmid library along with a Bxb1 integrase expression plasmid into a HEK293T cell line that (i) harbors a landing pad for single-copy, site-specific integration and (ii) expresses the reverse tetracycline transactivator (rtTA) for doxycycline (dox)-inducible activation of the TetOn promoter that drives expression of RHO variants fused to a transcription factor (TF) via a TEV protease-cleavable linker. For the assay, cells are expanded and RHO expression is induced with dox. A TEV protease anchored to the plasma membrane via the Lyn11 domain cleaves the linker between properly trafficked RHO and the TF, allowing for the TF to translocate to the nucleus, bind a TF upstream activating sequence (UAS), and induce expression of a reporter gene with a *RHO* variant-specific RNA barcode. Cells are lysed, RNA is purified, and barcode RNA is selectively reverse transcribed and amplified with PCR. Libraries are subjected to NGS, statistical modeling is performed on the sequencing counts to infer variant effects, and data are further analyzed and visualized. Created in BioRender. Manian, K. (2026) https://biorender.com/c58w2z0

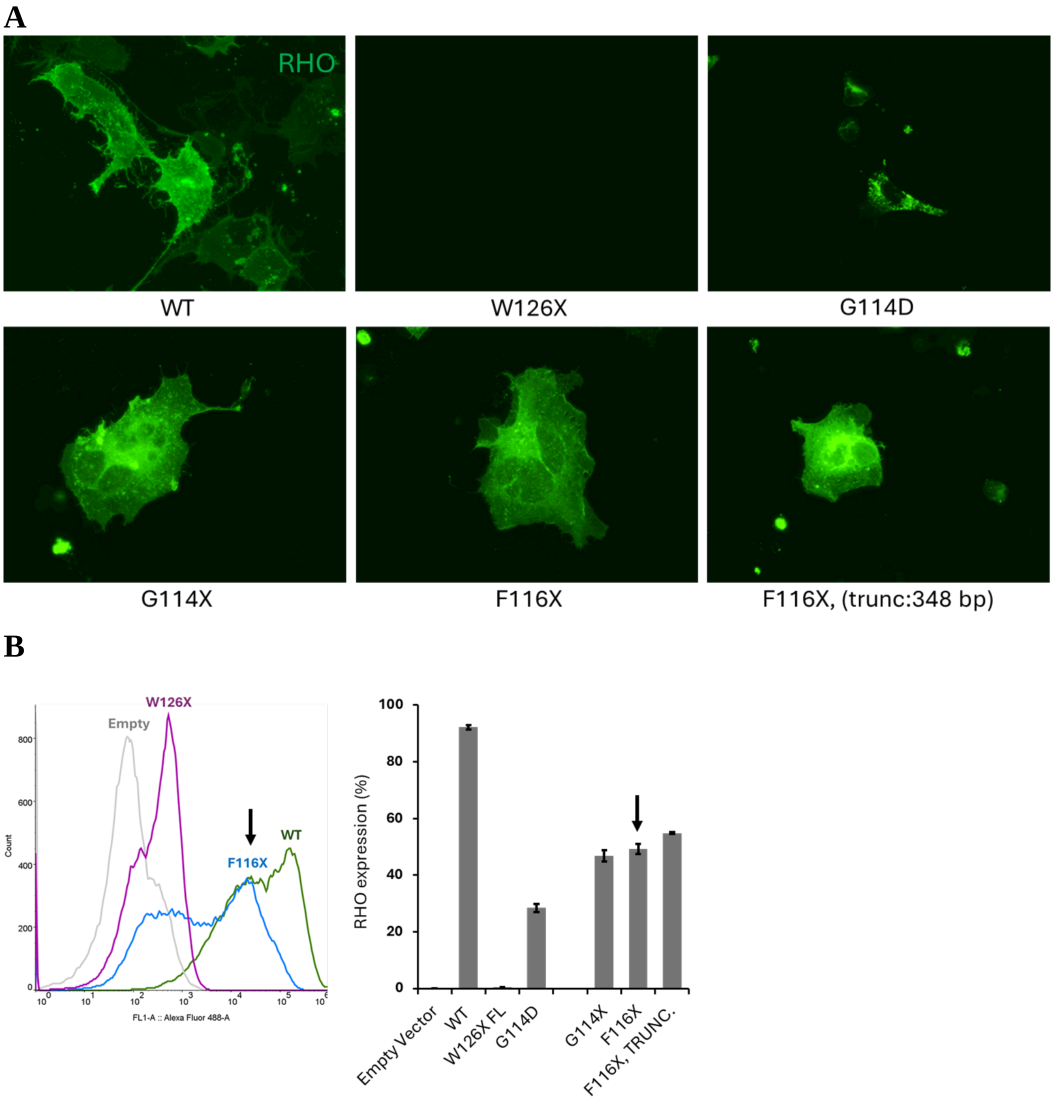

Figure S2. Nonsense variants between residues 110-125 show surface localization in transfected HEK293T cells. In contrast to WT rhodopsin, nonsense variants in the DMS library categorized as 'X' (in the heatmap) were predicted to have low surface trafficking scores. However, nonsense variants in the amino acid sequence between positions 110-125 exhibit intermediate-to-high surface trafficking scores in Method 1. To further investigate these findings, two nonsense variants from this region, and appropriate controls, were transfected and characterized by immunofluorescence and flow cytometry. In this experiment, nonsense variants G114X, F116X, and W126X (designed to have full-length cDNA sequences) and a nonsense variant F116X trunc. (with a shorter cDNA, with sequence termination after the stop codon) were used. **A)** Fluorescence microscopy images showing surface localization of RHO protein, with G114X, F116X, F116X truncated variants, alongside wild-type, in transfected HEK293T cells. In contrast, a nonsense variant W126X fails to form functional protein due to a PTC (premature termination codon), and a known class II misfolding variant G114D shows low surface expression. **B)** Flow cytometry analysis indicating nonsense variants G114X, F116X, and F116X (trunc.) with intermediate RHO cell surface trafficking. Wild-type RHO served as a positive control, exhibiting robust trafficking. In contrast, W126X displayed no detectable surface expression, while G114D showed reduced

surface expression (n=3 replicates). Both assays were performed on non-permeabilized cells. (Empty vector = backbone vector with no insert, WT = Wild-type rhodopsin, G114X, F116X, and F116X (trunc.) = a nonsense variant with surface trafficking, W126X = a nonsense variant with no surface trafficking, G114D = a class II variant.

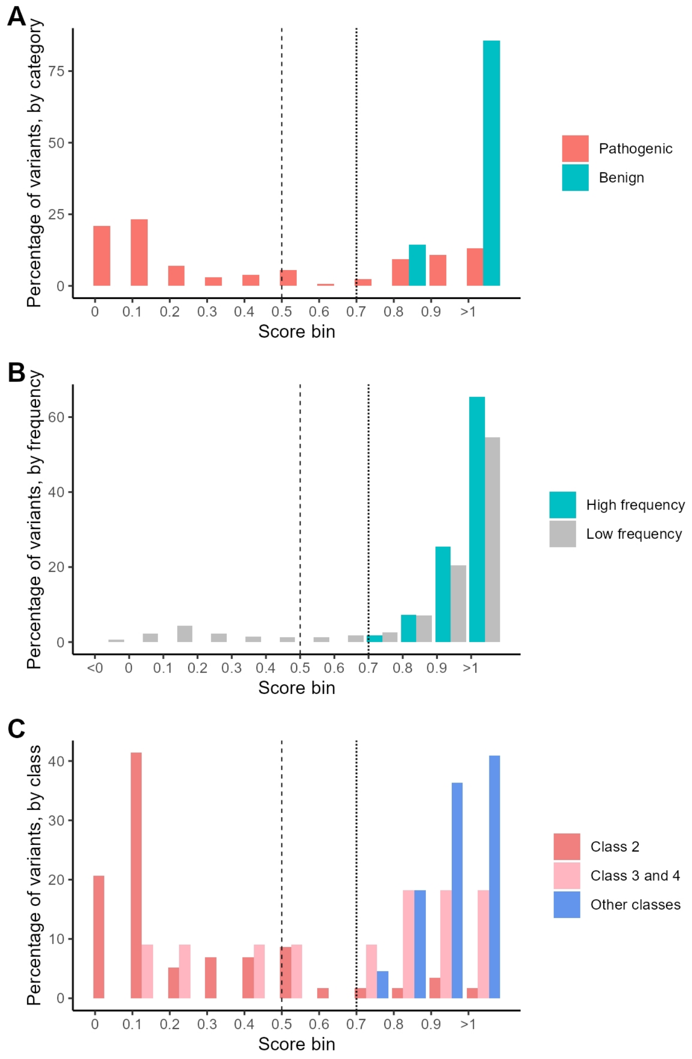

Figure S3. Investigating pathogenicity cutoff values. To determine which trafficking scores correspond to pathogenic variants, the ClinVar pathogenicity (top), gnomAD allele frequency (middle), or rhodopsin mechanistic classes (bottom) are shown as a function of trafficking score bins. Results are shown with normalization approaches: (**A**) as percentage of variants in each ClinVar category (Pathogenic/Benign), (**B**) Percentage allele frequency in gnomAD, or (**C**) percentage of variants in the mechanistic classes. There are no benign variants, high-frequency variants, or non-class 2,3,4 variants with a trafficking score < 0.7 (vertical dotted line), which defined the cutoff for moderate confidence mistrafficking variants. A cutoff for high confidence mistrafficking variants was set at 0.5 (vertical dashed line) because the underlying data is sparse (top: 7 of 136 variants are benign; bottom: 22 of 144 variants belong to the Other classes).

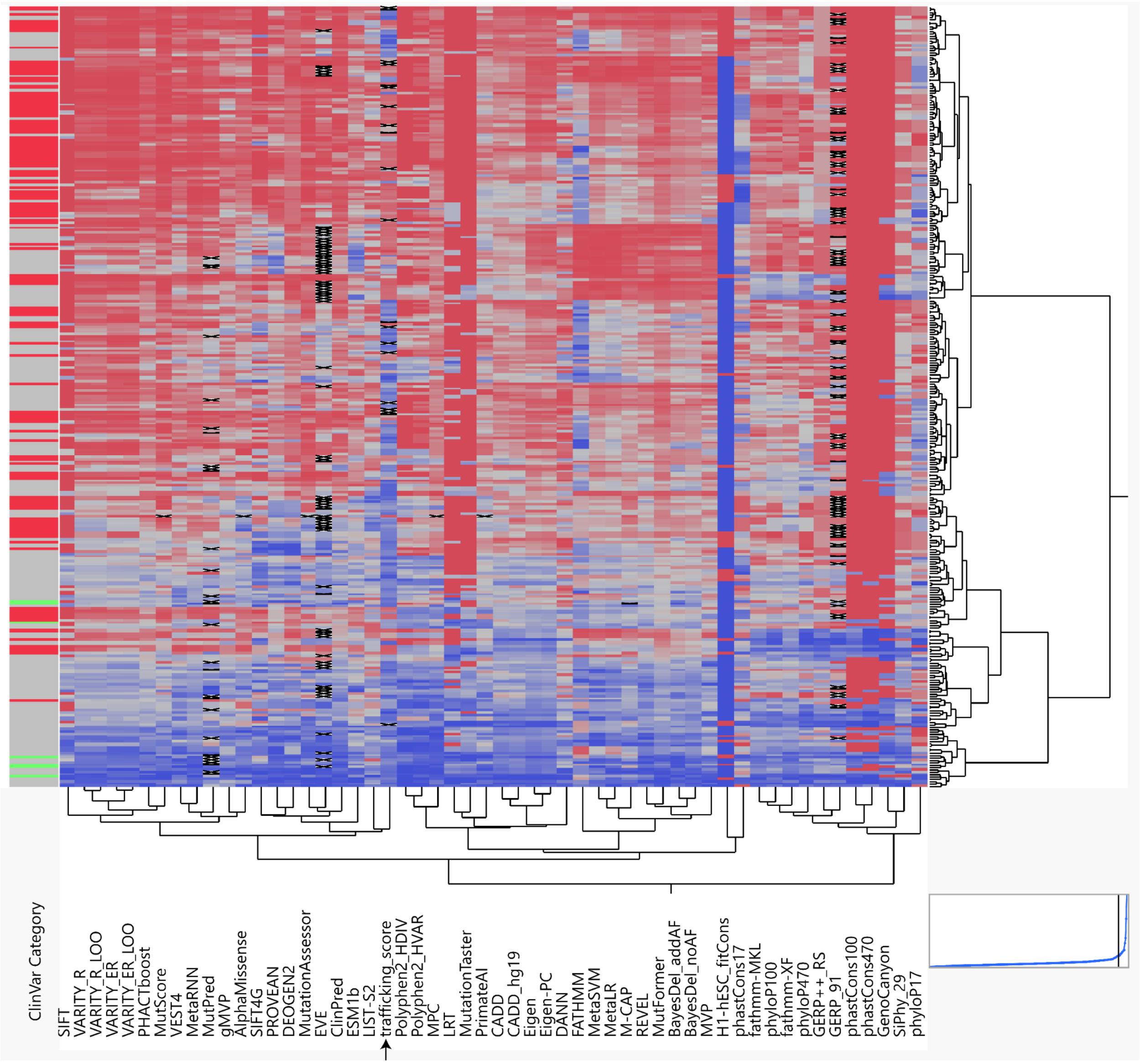

Figure S4. Comparison of trafficking scores with computational predictors of pathogenicity. (A) Hierarchical clustering groups variants (rows) and predictors (columns) that display similar patterns. Blue (0) represents a benign prediction while Red (1) represents a pathogenic prediction. On the left, the ClinVar Category is displayed; Red for pathogenic variants, Grey for variants of uncertain significance (VUS) and Green for benign variants. The trafficking score (up-pointing arrow) has the longest dendrogram branch, indicating it is the most distinctive predictor. Hierarchical clustering was performed using the Ward method with robustly normalized columns, missing value imputation (black), and with row clusters ordered by the first principal component of the data (JMP software v17.2).
